# Evolutionary analysis and functional characterization of SiBRI1 as a Brassinosteroid receptor gene in foxtail millet

**DOI:** 10.1186/s12870-021-03081-8

**Published:** 2021-06-24

**Authors:** Zhiying Zhao, Sha Tang, Yiming Zhang, Jingjing Yue, Jiaqi Xu, Wenqiang Tang, Yanxiang Sun, Ruiju Wang, Xianmin Diao, Baowen Zhang

**Affiliations:** 1 Ministry of Education Key Laboratory of Molecular and Cellular Biology, Hebei Collaboration Innovation Center for Cell Signaling and Environmental Adaptation, Hebei Key Laboratory of Molecular and Cellular Biology, College of Life Sciences, Hebei Normal University, Shijiazhuang, Hebei, 050024 China; 2 Institute of Crop Sciences, Chinese Academy of Agricultural Sciences, Beijing, 100081 China; 3 College of Life Sciences, Langfang Normal University, Langfang, 065000 China; 4 Foxtail Millet Improvement Center of China, Institute of Millet Crops, Hebei Academy of Agricultural and Forestry Science, Shijiazhuang, 050031 China

**Keywords:** Brassinosteroids, BRI1, Foxtail millet, Phylogenetic analysis

## Abstract

Brassinosteroids (BRs) play important roles in plant growth and development. Although BR receptors have been intensively studied in *Arabidopsis*, those in foxtail millet remain largely unknown. Here, we show that the BR signaling function of BRASSINOSTEROID INSENSITIVE 1 (BRI1) is conserved between *Arabidopsis* and foxtail millet, a new model species for C4 and Panicoideae grasses. We identified four putative BR receptor genes in the foxtail millet genome: *SiBRI1*, *SiBRI1-LIKE RECEPTOR KINASE 1* (*SiBRL1*), *SiBRL2* and *SiBRL3*. Phylogenetic analysis was used to classify the BR receptors in dicots and monocots into three branches. Analysis of their expression patterns by quantitative real-time PCR (qRT-PCR) showed that these receptors were ubiquitously expressed in leaves, stems, dark-grown seedlings, roots and non-flowering spikelets. GFP fusion experiments verified that SiBRI1 localized to the cell membrane. We also explored the SiBRI1 function in *Arabidopsis* through complementation experiments. Ectopic overexpression of SiBRI1 in an *Arabidopsis* BR receptor loss-of-function mutant, *bri1-116*, mostly reversed the developmental defects of the mutant. When SiBRI1 was overexpressed in foxtail millet, the plants showed a drooping leaf phenotype and root development inhibition, lateral root initiation inhibition, and the expression of BR synthesis genes was inhibited. We further identified BRI1-interacting proteins by immunoprecipitation (IP)-mass spectrometry (MS). Our results not only demonstrate that SiBRI1 plays a conserved role in BR signaling in foxtail millet but also provide insight into the molecular mechanism of SiBRI1.

**Supplementary Information:**

The online version contains supplementary material available at 10.1186/s12870-021-03081-8.

## Background

Foxtail millet (*Setaria italica*) is a diploid C4 panicoid crop. Because of its remarkable drought and stress tolerance, high water use efficiency, and excellent nutritional value, foxtail millet is one of the most important arid and semiarid land crops in the world [1]. The sequencing of the foxtail millet genome (423 Mb) was completed in 2012 [2], which laid a foundation for our further study on the agronomic traits and biological and abiotic stresses of foxtail millet. 

Brassinosteroids (BRs) are a group of natural polyhydroxy steroids that regulate diverse physiological processes in plants, including growth promotion, skotomorphogenesis, organ boundary formation, stomatal development, sex determination, vascular differentiation, male fertility, seed germination, flowering, senescence, and resistance to various abiotic and biotic stresses [3–5]. BRs are recognized by a membrane-localized LEUCINE (Leu)-RICH REPEAT (LRR) RECEPTOR-LIKE KINASE (RLK), BRI1, and its coreceptor BRI1-ASSOCIATED RECEPTOR KINASE 1 (BAK1). BR binding promotes the association of BRI1 with BAK1 and enables transphosphorylation between the cytoplasmic kinase domains of the two receptors [6, 7]. BRI1 then phosphorylates two types of membrane-localized RECEPTOR-LIKE CYTOPLASMIC KINASEs (RLCKs), BR SIGNALING KINASEs (BSKs) [8] and CONSTITUTIVE DIFFERENTIAL GROWTH 1 (CDG1) [9], leading to activation of the protein phosphatase BRI1 SUPPRESSOR 1 (BSU1). BSU1 dephosphorylates and inhibits GSK3/Shaggy-like kinase BR INSENSITIVE 2 (BIN2) [10]. In the absence of BRs, BIN2 phosphorylates transcription factors BRASSINAZOLE RESISTANTs (BZRs) family, preventing them from regulating the transcription of downstream target genes [11]. BR signaling inhibits BIN2 and allows BZR1 to be dephosphorylated by PROTEIN PHOSPHATASE 2A (PP2A) [12]. Together with their binding partners, dephosphorylated BZR1-family transcription factors bind to BR response elements or E-box cis-elements and regulate the expression of many BR-responsive genes [13] (Figure S1). However, there have been few studies on the BR signaling pathway in foxtail millet. Understanding BR signaling in foxtail millet might provide insights to enable the yield of this important crop to be improved.

AtBRI1, the most important BR receptor in *Arabidopsis*, has a membrane-localized signal peptide in the N-terminus, 25 LRR domains, and a 70-amino acid island between LRR XXI and LRR XXII, which is essential for the perception of BRs [14]. Mutation of AtBRI1 in *Arabidopsis* leads to dwarf plants with small curled dark green leaves, photomorphogenesis in the dark, insensitiveness to exogenous BL treatment, accumulation of endogenous BRs, and feedback regulation of BR biosynthesis gene expression [15]. As an important receptor in the BR signaling pathway, BRI1 orthologous genes play critical roles in both the monocots and dicots, including *Arabidopsis*, *Zea mays*, *Lycopersicon esculentum*, *Glycine max*, *Medicago truncatula*, *Pisum sativum*, *Oryza sativa*, *Brachypodium distachyon*, and *Hordeum vulgare* [16–18]*.* Notably, GmBRI1 and MtBRI1 can rescue the weak mutant allele of AtBRI1, *bri1-5*, in *Arabidopsis* [15, 16, 19]*.* In maize and *B. distachyon*, RNAi-mediated knockout of BRI1 and its homologous gene results in a BR-insensitive dwarf phenotype [18, 20]. Mutations in BRI1 orthologues cause similar pleiotropic phenotypes in pea, tomato, rice and barley [21–24]. These results indicated that the functional conservation of BRI1 among different species. Previous work has demonstrated that DROOPY LEAF1 (DPY1) participates in BR signaling and inhibits the interaction between SiBRI1 and SiBAK1 [25], but SiBRI1 in foxtail millet has not yet been characterized.

To obtain insights into the functions of BRs in the C4 model species foxtail millet, we conducted an evolutionary and functional examination of foxtail millet BR receptors. We identified four putative BR receptor genes in the foxtail millet genome, *SiBRI1*, *SiBRL1*, *SiBRL2* and *SiBRL3*, and analysed their expression patterns and roles in the BR signaling pathway. Our findings showed that SiBRI1 could rescue the dwarf phenotype of *bri1-116*, and enhance the dephosphorylation of BZR1 in vivo to activate the BR signaling pathway. When SiBRI1 was overexpressed in foxtail millet, the plants showed droopy leaves, root development inhibition, and the expression of BR synthesis genes was inhibited. Furthermore, We also found 128 SiBRI1 interacting proteins through IP-MS, which laid the foundation for further study of SiBRI1 function.

## Results

### Phylogenetic analysis of BRI1 family genes

We investigated whether the foxtail millet genome encodes BR receptors of the canonical BR signal transduction pathway. We identified foxtail millet BR receptors by using the AtBRI1 and OsBRI1 protein sequences in Phytozome and the *Setaria italica* Functional Genomics Database with the BLASTP algorithm [26]. We found four putative BR receptor genes in the foxtail millet genome, which were named SiBRI1, SiBRL1, SiBRL2 and SiBRL3 (Table S1).

**Fig. 1:**
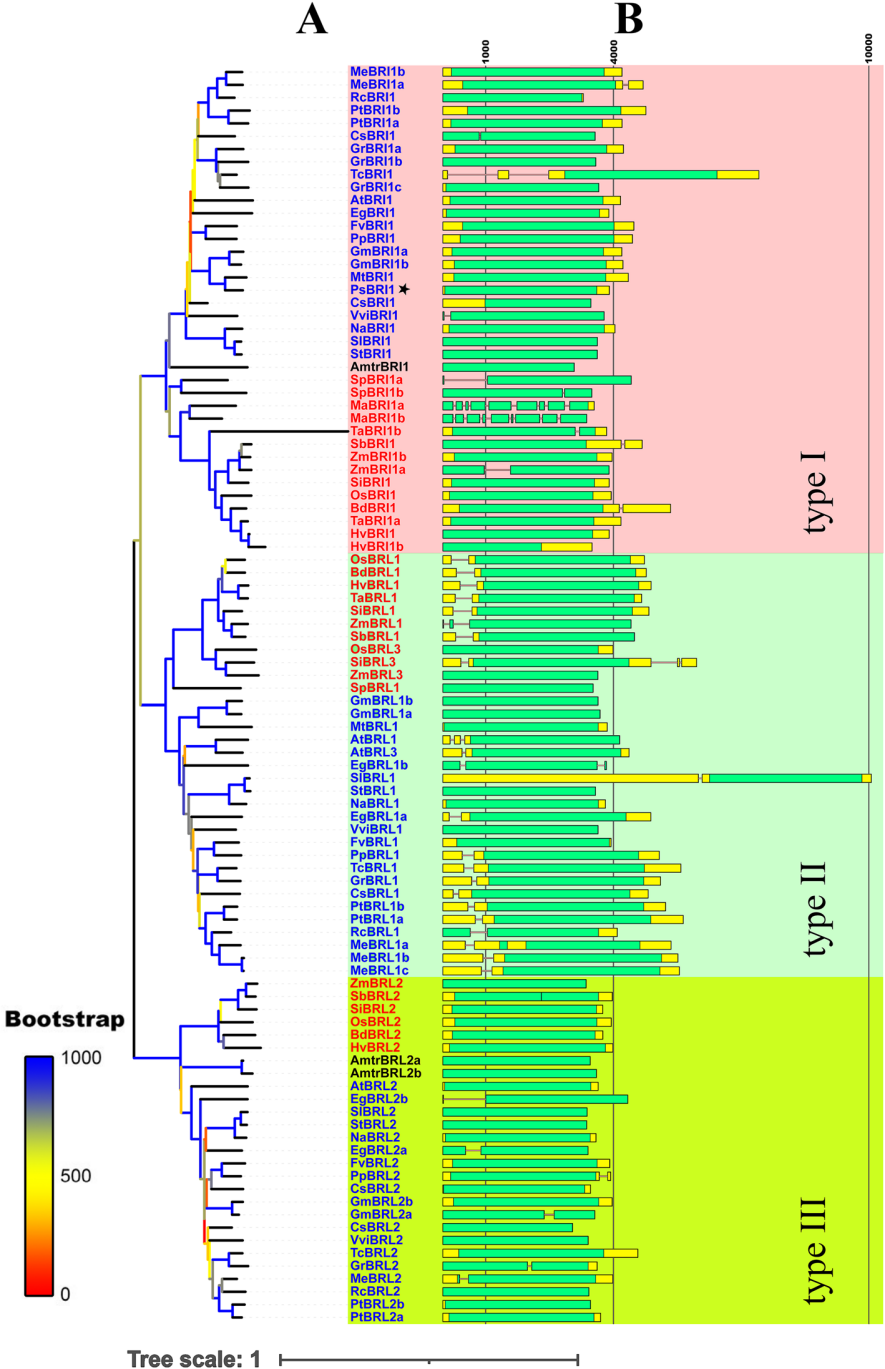
Phylogenetic evolutionary tree and gene structures of BRI1 gene family members. (**A**), an neighbor-joining (NJ) phylogenetic tree was constructed using the full-length protein sequence alignments of BRI1 genes identified using MUSCLE in MEGAX. Bootstrap supports were indicated by the colour of the branch. The OTUs are labelled as follows: Dicotyledons (blue); Monocotyledons (red); Amborella trichopoda (black). The colour blocks indicate the types, with type I (pink), type II (green), and type III (yellow) denoted. (**B**), Gene structures of the BRI or BRL genes. The lengths of rectangles and lines are scaled according to the mRNA lengths. CDSs (green rectangles), UTRs (yellow rectangles), and introns (black line) are denoted

We performed a phylogenetic analysis including SiBRI family members, the published BRI family genes (Table S1), and the representative plants that had relatively complete annotated genome data and phylogenetic relationship information by the Angiosperm Phylogeny Group (APG) taxonomy [27] (Figure S2). Because BRI1 belongs to the RLK superfamily, searching with a single hidden Markov model (HMM) cannot exclude the considerable redundancy. Therefore, the published BRI1 protein sequences were used to construct an HMM, and HMMER V 3.3 [28] was used to search for candidate genes with the complete protein sequence data of the species that hadn’t indentified the BRIs family genes before. Notably, we found BRI1 family genes only in angiosperms. Finally, 98 BRI1 gene family members were identified and used for phylogenetic analysis. To map the phylogenetic relationships among these members, MUSCLE alignment methods and neighbour-joining (NJ) phylogenetic inference methods were employed. In addition, the full-length BRI1 sequences were analysed separately (Fig. 1).

The plant BRI1 gene family members identified in the present study (Fig. 1A) could be divided into three subgroups: type I, type II and type III. The type I BRI1 family gene subgroup was the largest branch and accounted for 38.8% of the genes observed, the type II subgroup accounted for 33.7% of the genes, and the type III subgroup accounted for the remaining 27.5% of the genes. BRI1 belonged to type I, BRL1 and BRL3 belonged to type II, and BRL2 belonged to type III. The predominance of type I BRI1 genes may be related to the fact that these genes help regulate the whole process of plant growth and development, while BRL1 and BRL3 genes function mainly in the root [29]. Notably, BRL1 and BRL3 belong to the same type, while BRL2 belongs to a separate type. This result corresponds to the finding that the extracellular domain of BRL2 cannot interact with BR. In addition, the monocots and dicots were further divided into two clusters in each type (Fig. 1A).

SiBRI1 was predicted to have 53.96% identity with AtBRI1 and 79.40% identity with OsBRI1, its homologues in *Arabidopsis* and rice. Besides OsBRI1, ZmBRI1b, SbBRI1, ZmBRI1a, BdBRI1, HvBRI1, TaBRI1a, HvBRI1b belonged to a sub-branch of the phylogenetic tree with SiBRI1, and the homology is 86.8%, 86.7%, 83.2%, 81.3%, 81.5%, 81.5%, 80.3%, respectively. The protein sequence lengths of the BRI1 genes varied from 827 aa (CsBRI1 in *Cucumis sativa*) to 1288 aa (ZmBRL1 in maize), and most BRI1 genes had no introns (Fig. 1B). After cloning and sequencing of the flanking regions of SiBRI1 in *Setaria italica* (cultivar Yugu-1) with specific primers, the full-length cDNA was predicted to contain a long open reading frame that encoding a protein of 1118 aa. Alignment analysis indicated that SiBRI1 shares a conserved signal peptide, LRR, transmembrane (TM) domain and kinase domain with AtBRI1 and OsBRI1, and the critical amino acids for the kinase activity of AtBRI1 were also conserved in SiBRI1, suggesting that SiBRI1 is an active kinase (Figure S3).

### Tissue and subcellular localization of SiBRI1 and its orthologues

**Fig. 2:**
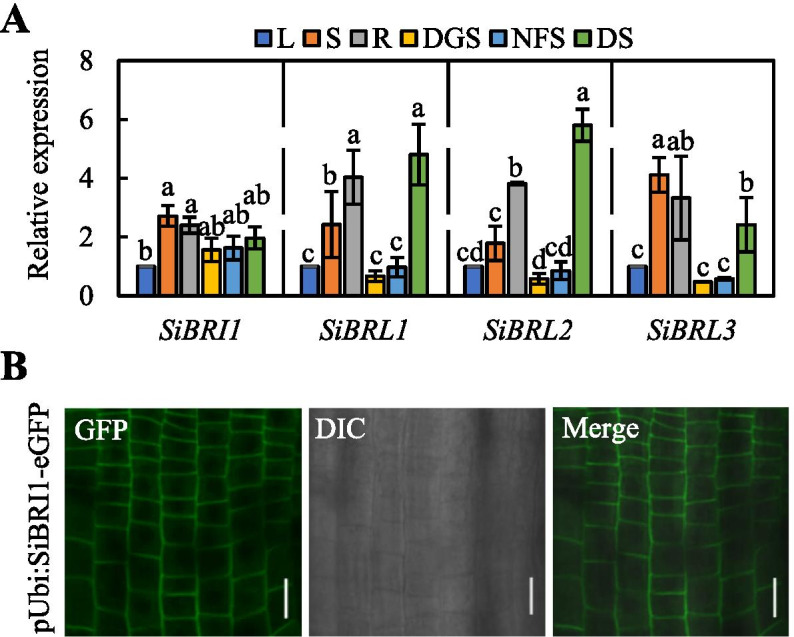
Expression and subcellular localization analysis of SiBRI1 and its orthologues in different tissues in foxtail millet. (**A**), Leaves (L), stems (S), dark-grown seedlings (DGS), roots (R), non-flowering spikelets (NFS), and dry seeds (DS). Error bars indicate the mean ± standard deviation (SD). *N* = 3. Statistically significant differences are indicated by different lowercase letters (*p* < 0.05, one-way ANOVA with Tukey’s significant difference test). (**B**), Confocal images indicate the localization of SiBRI1-eGFP in the roots of 3-day-old dark-grown seedlings overexpressing SiBRI1 with a GFP tag at the C-terminus. Scale bar = 20 µm

We used quantitative real-time PCR (qRT-PCR) to determine the expression patterns and transcript abundance of SiBRI1 and its orthologues in foxtail millet. SiBRI1 was universally expressed in the leaves (L), stems (S), dark-grown seedlings (DGS), roots (R), non-flowering spikelets (NFS), and dry seeds (DS) of foxtail millet (Fig. 2A). SiBRL1, SiBRL2 and SiBRL3 were highly expressed in root, stems and dry seeds (Fig. 2A). We speculate that SiBRI1 and its paralogues genes may play different roles in different tissues.

As mentioned above, a signal peptide in the N-terminus and a TM domain were predicted to be present in SiBRI1 (Figure S3). To determine the subcellular localization of SiBRI1, we constructed a fusion protein, SiBRI1::eGFP, driven by a maize ubiquitin promoter and transformed it into foxtail millet. Then, we detected clear fluorescence signals on the plasma membrane in root meristem cells by laser confocal microscopy (Fig. 2B). The results showed that SiBRI1 is a cell membrane protein.

### Functional analysis of SiBRI1 in *Arabidopsis*

**Fig. 3:**
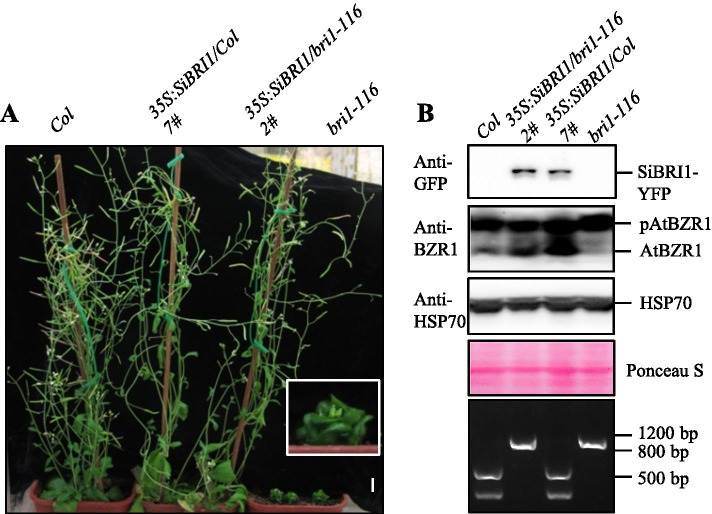
SiBRI1 overexpression rescued the mutant phenotypes of *bri1-116* plants. (**A**), Phenotypes of light-grown 8-week-old *Col-0* and *bri1-116* mutant plants overexpressing SiBRI1 with a C-terminal YFP tag. An enlarged view of *bri1-116* is shown in the white box. Scale bar = 1 cm. (**B**), Expression levels of SiBRI1-YFP and AtBZR1 in the transgenic plants shown in (**A**). The differential accumulation pattern of AtBZR1 in (**A**) was detected by anti-BZR1; pAtBZR1 showed the phosphorylation form of AtBZR1, and AtBZR1 showed the unphosphorylated form of AtBZR1. Anti-HSP70 and Ponceau S staining of the Rubisco large subunit was used as an equal loading control. The bottom gel shows the genotyping identification of the transgenic plants in (**A**)

To verify whether SiBRI1 encodes a BR receptor, we transformed the SiBRI1-encoding sequence under the control of the CaMV 35S promoter into *Arabidopsis Columbia-0* (*Col-0*) and a BR-insensitive stunted *Arabidopsis* mutant, *bri1-116*. *bri1-116* is produced by a point mutation of Glutamate at site 583 in the 21st LRR before the AtBRI1 island domain. This mutation results in early termination of the peptide chain and a phenotype with complete AtBRI1 deletion, severe plant dwarfism, shortened petioles, and shrunken and rounded leaves [14]. After SiBRI1 overexpression, the plant height, silique length, and leaf blade morphology in the *bri1-116* (SiBRI1/*bri1-116*) transgenic lines were observed to be similar to *Col-0*, and the leaf curling and elongation exhibited by BR-activated plants were observed after SiBRI1 overexpression in *Col-0* (SiBRI1/*Col*) plants (Fig. 3A and S4). Previous studies have shown that when BR levels are low, BIN2 phosphorylates and inactivates BZR1 to inhibit plant growth; BRs promote growth by inducing the dephosphorylation of BZR1, a hallmark of active BR signaling pathway [11]. Therefore, we detected the phosphorylation levels of AtBZR1 in different transgenic plants. In *Col-0*, a weak band was observed for unphosphorylated AtBZR1, whereas in *bri1-116*, the band for unphosphorylated AtBZR1 was almost undetectable. When SiBRI1 was transferred into the *Col-0* background or into *bri1-116*, the strength of the band for unphosphorylated AtBZR1 increased significantly (Fig. 3B), indicating that the BR signal had been activated.

**Fig. 4:**
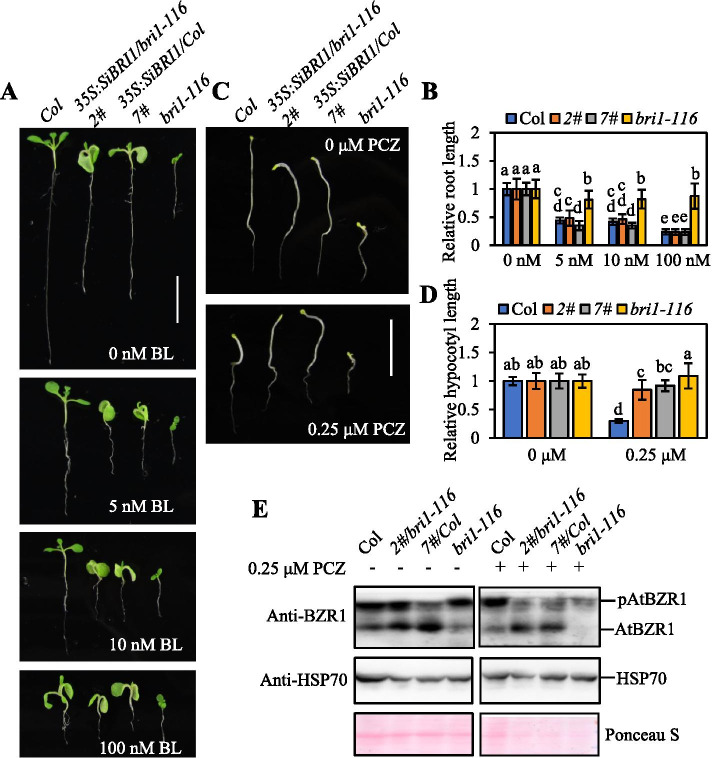
SiBRI1 overexpression activated BR signaling in *bri1-116* plants. (**A**), Phenotypes of *Col-0* and *bri1-116* mutant plants overexpressing SiBRI1 that were grown in the presence of the indicated concentration of BL for 7 days. Scale bar = 1 cm. (**B**), Relative root lengths of the plants in (**A**). Error bars indicate the mean ± standard deviation (SD). N > 30. Statistically significant differences are indicated by different lowercase letters (*p* < 0.05, two-way ANOVA with Tukey’s significant difference test). (**C**), Phenotypes of *Col-0* and *bri1-116* mutant plants overexpressing SiBRI1 that were grown in the presence of the indicated concentration of PCZ for 7 days. Scale bar = 1 cm. (**D**), Relative hypocotyl lengths of the plants in (**C**). Error bars indicate the mean ± standard deviation (SD). N > 30. Statistically significant differences are indicated by different lowercase letters (*p* < 0.05, two-way ANOVA with Tukey’s significant difference test). (**E**), Immunoblot analysis of BZR1 in transgenic plants overexpressing SiBRI1 in the *bri1-116* mutant or wild type background under PCZ. pAtBZR1 showed the phosphorylation form of AtBZR1, and AtBZR1 showed the unphosphorylated form of AtBZR1. Expression levels of HSP70 and AtBZR1 in the transgenic plants shown in (**C**). Ponceau S staining of the Rubisco large subunit and the expression level of HSP70 was used as an equal loading control

Exogenous application of low levels of bioactive brassinolide (BL) promotes plant growth. To examine the responsiveness of SiBRI1 overexpression seedlings to exogenous BL application, we grew seeds on ^1^/_2_ Murashige and Skoog (MS) medium supplemented with BL at a series of concentrations (0, 5, 10 and 100 nM) for seven days and measured the primary root length. Without BL treatment, the root length of SiBRI1/*bri1-116* was significantly greater than that of *bri1-116* but approximately 38% less than that of *Col-0*, and the root length of SiBRI1/*Col* was shorter than that of *Col-0*. SiBRI1 /*Col* exhibited a phenotype of slightly shorten primary roots, similar to that generated following BR application, as BR treatment inhibited the lengthening of plant roots (Fig. 4A). SiBRI1/*Col* seedlings grown on ^1^/_2_ MS media containing 5 and 10 nM BL showed greater sensitivity to BL than *Col-0* seedlings, and SiBRI1/*bri1-116* plants showed greater sensitivity to BL than *bri1-116* plants under 5, 10 and 100 nM BL treatment in a concentration dependent manner (Fig. 4B). These results indicated that the SiBRI1 over expression plants exhibited enhanced sensitivity to BL.

Propiconazole (PCZ) is a specific inhibitor of BR biosynthesis and inhibits hypocotyl elongation under both light and dark conditions in *Arabidopsis* and maize [30]. To determine the responses of SiBRI1 over expression seedlings to PCZ, we cultivated germinated seeds on ^1^/_2_ MS medium containing 0.25 µM PCZ in the dark for seven days. Under dark conditions, 0.25 µM PCZ decreased the hypocotyl length of wild-type seedlings by 67%. The hypocotyls of SiBRI1 over expression seedlings had a wavy, twisted phenotype. The hypocotyl length of *bri1-116* seedlings was very short and showed no significant difference between the PCZ and non-PCZ treatments. SiBRI1/*bri1-116* and SiBRI1/*Col* seedlings showed greater insensitivity to PCZ than *Col-0* seedlings (Fig. 4C, D, and S4). We also tested the phosphorylation levels of AtBZR1 in different transgenic plants and found that the phosphorylation of AtBZR1 in the transgenic plants was inhibited (Fig. 4E). All these results indicate that the SiBRI1 transgene successfully complements the BR-insensitive phenotype in *bri1-116*.

### SiBRI1 regulates BR signaling in foxtail millet

**Fig. 5:**
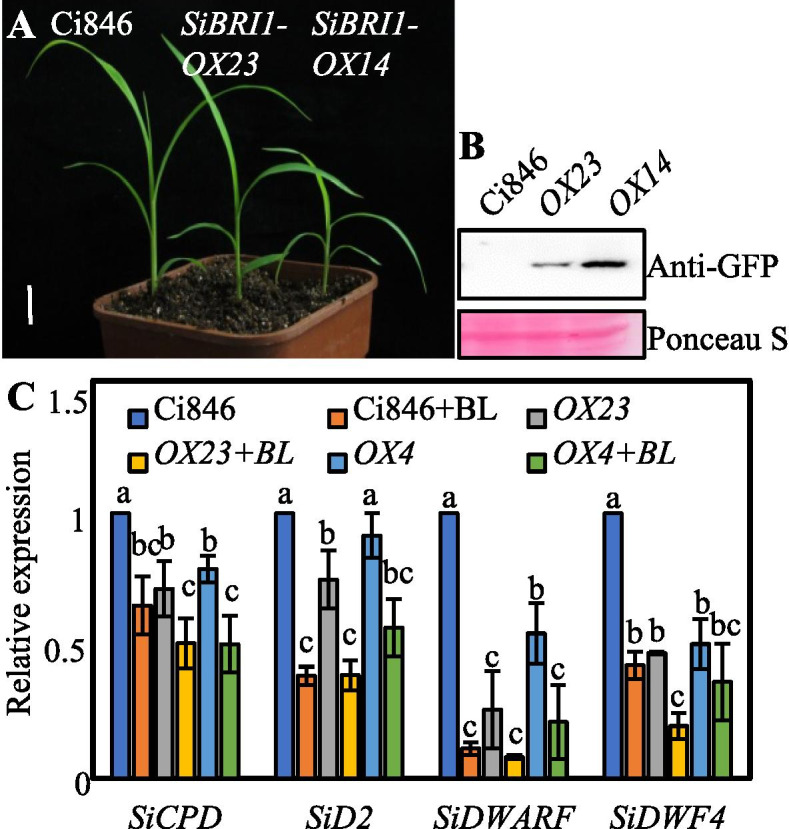
SiBRI1 regulated the BR response in foxtail millet. (**A**), Phenotype of light-grown 4-leaf stage Ci846 plants and two independent lines of *pUbi:SiBRI1-eGFP/*Ci846 (*SiBRI1-OX*); scale bar = 1.5 cm. (**B**), Expression levels of SiBRI1-YFP in the transgenic plants shown in (**A**). Ponceau S staining of the Rubisco large subunit was used as an equal loading control. (**C**), Quantitative real-time RT-PCR analysis of *SiCPD*, *SiD2*, *SiDWARF* and *SiDWF4* expression in the roots of 8-day-old seedlings overexpressing SiBRI1 under 1 μM BL immersion for 1 h. Three biological repetitions were established. Error bars indicate the mean ± standard deviation (SD). Statistically significant differences are indicated by different lowercase letters (*p* < 0.05, one-way ANOVA with Tukey’s significant difference test)

To test whether SiBRI1 regulates BR signaling in foxtail millet, we overexpressed full-length SiBRI1 in Ci846 which is an easily transformed variety of *S. italica* and generated a pUbi:SiBRI1-eGFP transgenic (*SiBRI1-OX*) plant through callus transformation. At the 4-leaf stage, *SiBRI1-OX* seedlings in two independent lines, *OX23* and *OX14*, had larger leaf angles and droopier leaves than Ci846 seedlings (Fig. 5A); notably, large leaf angles and drooping leaves have been reported to be specific phenotypes controlled by BR in foxtail millet [25]. Both of these phenotypes were dependent on the protein expression level of SiBRI1 (Fig. 5B). Notably, the transcript levels of SiBRI1 were downregulated after BR treatment in both leaves and roots (Figure S5), consistent with our published RNA-Seq data [31].

Activation of the BR signaling pathway is known to inhibit the expression of BR synthesis genes via a feedback mechanism. To verify that SiBRI1 is the receptor of BR, a qRT-PCR assay was used to detect the responses of the BR synthesis genes *SiCPD*, *SiD2*, *SiDWARF* and *SiDWF4* in the roots of different *SiBRI1-OX* transgenic lines in Ci846 (Figure S6). The qRT-PCR results showed that the transcript levels of these genes were reduced by BR regulation and that the expression of these genes was lower in *SiBRI1-OX* plants than in Ci846 plants (Fig. 5C). Taken together, these results indicate that SiBRI1 overexpression activates the BR signaling pathway in foxtail millet and that SiBRI1 is a receptor in the conserved BR signaling pathway.

### SiBRI1 affects root growth and lateral root development in foxtail millet

**Fig. 6:**
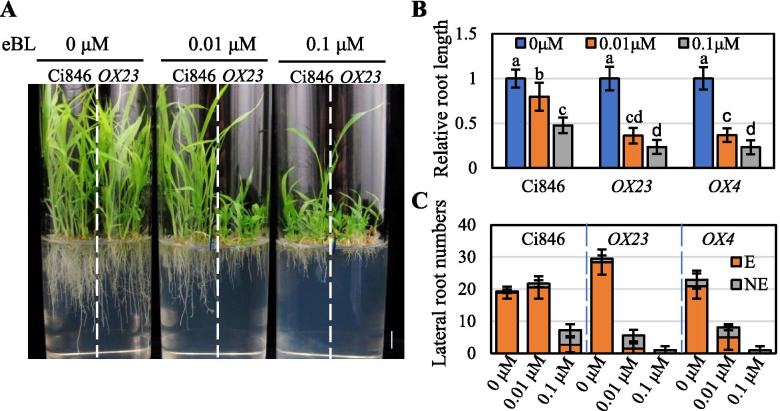
*SiBRI1-OX* was hypersensitive to BL in foxtail millet. (**A**), Phenotypes of Ci846 plants overexpressing SiBRI1 that were grown in the presence of the indicated concentration of BL for 6 days under 16L/8D 28 °C. Bar = 1 cm. (**B**), Relative root lengths of the plants in (**A**). N > 12. Error bars indicate the mean ± standard deviation (SD). Statistically significant differences are indicated by different lowercase letters (*p* < 0.05, two-way ANOVA with Tukey’s significant difference test). (**C**), The lateral root numbers of seedlings in (**A**). N > 12. Lateral roots with lengths greater than 1 mm are marked as elongated (**E**), those with lengths less than 2 mm are marked as unelongated (NE)

As BRI1 is a vital positive modulator in the BR signaling pathway, *BRI1-OX* plants are sensitive to the BL growth response, i.e., the inhibition of root development in *Arabidopsis* and rice [32], as described above. To determine whether SiBRI1 similarly plays a positive regulatory role in foxtail millet, we investigated the responses of two independent *SiBRI1-OX*/*Ci846* lines differing in their expression levels of SiBRI1 to 0.01 and 0.1 µM BL (Fig. 6A and S6). BL at a concentration of 0.01 µM significantly inhibited root and leaf growth in *OX23* and *OX4* plants compared with wild-type plants. When the BL concentration was increased to 0.1 µM, leaf and primary root growth were also significantly inhibited in wild-type plants, but the inhibition was more obvious in *OX23* and *OX4* plants than in wild-type plants (Fig. 6A, B).

We also counted lateral roots in *SiBRI1-OX/Ci846* plants following treatment with different concentrations of BL. *SiBRI1-OX/Ci846* plants had more lateral roots than Ci846 plants (Fig. 6C). Seedlings grown on medium containing 0.01 µM BL showed short primary roots but had more lateral roots than control seedlings (no BL), whereas primary root length and lateral root number both decreased with increasing BL concentration in Ci846 plants (Fig. 6B-C). These results indicated that the sensitivity of lateral root development to the BR response differs from that of primary root elongation; specifically that primary root elongation is more sensitive than lateral root development to BL. However, the number of lateral roots in *SiBRI1-OX/Ci846* plants was drastically decreased and was much lower than that in control plants when the media contained 0.01 µM BL (Fig. 6C). Therefore, we concluded that the *SiBRI1-OX/Ci846* plants were more sensitive to exogenous BR than the control plants. These findings also support that BRI1 as a positive regulator of BR signal transduction in foxtail millet.

**Fig. 7:**
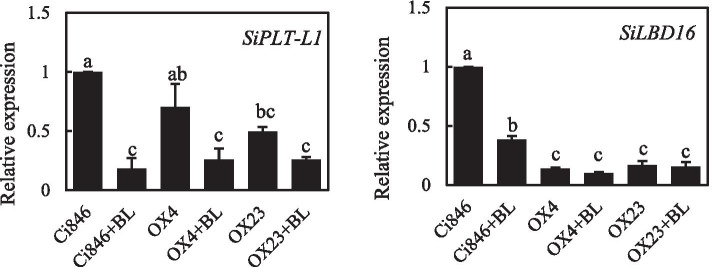
Quantitative RT-PCR analysis of *SiPLT-L1* and* SiLBD16* expression in the roots of 8-day-old *SiBRI1* overexpressing plants. The transgenic plants and Ci846’s roots were immersed under 1 μM BL for 1 h. Two–three biological repetitions. Error bars indicate the mean ± standard deviation (SD). Statistically significant differences are indicated by different lowercase letters (*p* < 0.05, one-way ANOVA with Tukey’s significant difference test)

PLETHORA 1 (PLT1) in *Arabidopsis* play an important role in regulating root development [33], we found that the expression of *PLETHORA-LIKE 1* (*SiPLT-L1*, based on the homology with *AtPLTs*) in foxtail millet roots decreased after BR treatment, and the transcript level of *SiPLT-L1* in *SiBRI1-OX/Ci846* was significantly lower than Ci846 (Fig. 7). LATERAL ORGAN BOUNDARIES DOMAIN16 (LBD16) play pivotal role in lateral root initiation [34], we also test the transcription of *SiLBD16* (based on the homology with *AtLBD16*) under BL in the roots of *SiBRI1-OX/Ci846* and Ci846 by qRT-PCR, and found that *SiLBD16* significantly dropped under BL treatment, and the transcription of *SiLBD16* in SiBRI1-OX/*Ci846* was lower than Ci846, which was corresponding to the phenotype of *SiBRI1-OX/Ci846* under BL (Figs. 7 and 6C). In conclusion, BR may effect root growth and lateral root development via SiPLT-L1 and SiLBD16.

### The interaction proteins of SiBRI1 in foxtail millet

**Fig. 8:**
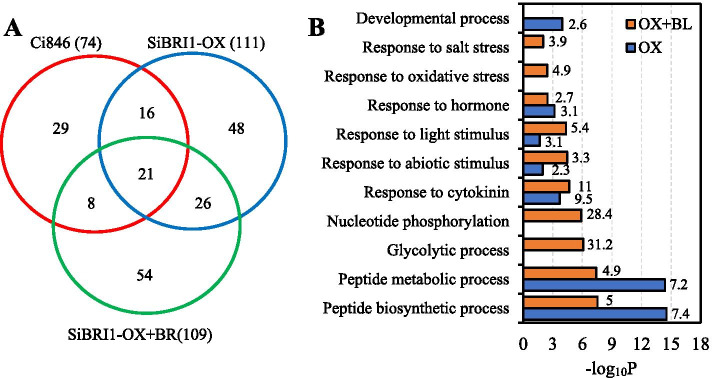
SiBRI1 interaction proteins under BL treatment. (**A**). The Venn diagram of the proteins identified in *pUbi:SiBRI1-eGFP*/Ci846 under BL treatment. 8-days-old Ci846 and *pUbi:SiBRI1-eGFP*/Ci846 seedlings was treated with 1 µM BL for 2 h and the overground tissues used to extract the interaction proteins with SiBRI1 by GFP-Trap. (**B**) GO annotation analysis of the SiBRI1 interaction proteins in *pUbi:SiBRI1-eGFP*/Ci846 under BL treatment or not, Gene ontology (GO) annotation was performed online using AgriGO (http://bioinfo.cau.edu.cn/agriGO/), the numbers in bar polots represent the fold enrichment compared with the whole-genome level

To understand the functions of SiBRI1 and the mechanisms underlying SiBRI1-mediated BR signaling specificity, we performed a proteomics study of SiBRI1-interacting proteins in foxtail millet. Microsomal proteins were extracted from the *SiBRI1-OX/Ci846* plants treated with BL or not, or the non-transgenic Ci846 as negative control, and SiBRI1-eGFP and associated proteins were immunoprecipitated using GFP-Trap. Liquid chromatography tandem mass spectrometry (LC–MS/MS) analysis identified 128 proteins that were co-immunoprecipitated in the SiBRI1-eGFP sample but not in the non-transgenic Ci846 control sample, 48 proteins that were co-immunoprecipitated from the *SiBRI1-OX/Ci846* plants without BL treatment, 54 proteins that were co-immunoprecipitated only from the *SiBRI1-OX/Ci846* plants with BL treatment, and 26 proteins that were co-immunoprecipitated from the *SiBRI1-OX/Ci846* plants regardless of BL treatment (Fig. 8A, Table S2-4).

Gene Ontology (GO) annotation and enrichment analysis was performed for the SiBRI1 interaction proteins with BL treatment or not separately. To analyse the effective information of proteins more accurately, GO annotation was performed online using AgriGO (http://bioinfo.cau.edu.cn/agriGO/). For the 74 interaction proteins that found in the *SiBRI1-OX/Ci846* plants without BL treatment, the proteins related with development, hormone, peptide metabobic and biosynthetic was enriched. When the BL treatment to the seedling, the SiBRI1 interaction proteins changed to related with nucleotide phosphorylation, glycolytic process, hormone, light stimulus, abiotic stimulus and so on (Fig. 8B, Table S5). We also found SiBSK7, a homologous gene of AtBSK7 in *Arabidopsis*, and BSKs has been reported to be the direct substrate of BRI1 in the BR signaling pathway [8]. In conclusion, our work lays a foundation for future studies on the function of SiBRI1 in foxtail millet.

## Discussion

Foxtail millet has helped nourish human civilizations throughout Asian history, and it is still considered a staple food today. Unlike other staple crops (e.g., rice, wheat, and maize), foxtail millet can grow in arid or semiarid areas where the water supply and soil fertility are limited. With the increasing impact of global warming and loss of arable land due to human activity, foxtail millet is an excellent substitute crop that can help secure food safety for human societies in the future. However, compared with other popular staple crops, the average yield of foxtail millet is low. This leaves great opportunity for future improvement by using modern inbreeding technology.

In general, leaf angle, effective tiller number, number of grains per panicle, and thousand-grain-weight are the most important traits that determine the average yields of cereal crops. Studies of rice indicate that BRs play an important role in regulating these productivity-related traits. Although BR signaling has been extensively studied in several species, BR signaling in foxtail millet is still largely uncharacterized.

Identifying BR receptors in more plants and deciphering their functions are important initial steps towards deciphering BR signaling networks and understanding their evolution. In this study, we cloned the foxtail millet BR receptor gene SiBRI1 and confirmed that it functioned as a BR receptor in *Arabidopsis* and foxtail millet at the physiological, genetic, and molecular levels. We also identified three other foxtail millet BR receptor genes, *SiBRL1*, *SiBRL2* and *SiBRL3*. Phylogenetic analysis was used to classify the BR receptors in angiosperms into three types: I (BRI1), II (BRL1 and BRL3) and III (BRL2). Our phylogenetic analysis also provides a basis for naming BRI1 family genes of other species in the future. BRI1 and its homologous genes play roles in different tissues in *Arabidopsis thaliana* and rice [21, 35]. Our qRT-PCR experiment revealed that the expression patterns of SiBRI1 and its paralogues genes differed in different tissues, indicating that these genes may play different roles in different tissues. Due to the importance of BRI1, the roles of BRI1 in regulating plants development has been commendable studied recently, the literature on the specific roles of BRLs are relatively scare. AtBRL3 has been reported to be involved in plant drought defense and ROS homeostasis [36, 37]. The study of BRLs involved in growth and development pathways in plants, improving the understanding of the specific roles of different BRI family genes.

To date, homologues of the BR receptor BRI1 have been identified and studied in five monocot species: rice, maize, barley, wheat and *B. distachyon* [18, 20, 21, 24, 38, 39]. The functions of the BRI1 homologues in *B. distachyon* and maize have been revealed in studies on plants in which BdBRI1 and ZmBRI1, respectively, were silenced by RNAi [18, 20]. In addition to OsBRI1, OsBRL1 and OsBRL3 have been characterized in rice. And different defective alleles of BRI1 showed a canonical BR-defective phenotype in rice and barley [21, 24].

However, prior studies on *B. distachyon* had failed to determine whether the monocot homologues of BRI1 are able to function as BR receptors in dicot plants [38]. Furthermore, in monocots, the end product of BR synthesis and the more active BR seems to be castasterone rather than BL. The structure of BRI1 was found to be conserved between monocot and dicot plants, so we hypothesized that the function of BRI1 was conserved in these plants. To test our hypothesis, we cloned the gene and heterologously expressed it in *bri1-116* plants. We found that the full-length coding sequence of SiBRI1 completely rescued the dwarf phenotype of *bri1-116. bri1-5* is a weak mutant allele of AtBRI1 containing a point mutation that results in a C69Y amino acid substitution in the extracellular domain of BRI1 [15]. Although BRI1 in some dicot plants, such as GmBRI1b, GmBRI1a and MtBRI1, has been demonstrated to restore the phenotype of *bri1-5* plants [16, 17, 19], whether the phenotype of *bri1-116*, the null allele of AtBRI1, can be resuced by ectopic expression is unclear. Our study proved that SiBRI1 could completely rescue the null allele of AtBRI1. These findings provide solid evidence that SiBRI1 can completely replace the function of AtBRI1. Our results demonstrate the conserved functions and structures of BRI1 between dicot and monocot plants and broaden our understanding of the BR response in crop plants.

The BR signaling pathway is both conserved and specific in different plants. Although we identified two key components involved in BR signaling in foxtail millet, SiBRI1 and SiBZR1 [31], the phenotypes after BR treatment differ between dicot and monocot plants. Dicot plants show increases in hypocotyl length and plant height, and reductions in root length under light conditions. For monocot plants, such as rice and maize, the main results of BR treatment are shortened roots and increased leaf angles [18, 32]; droopy leaves and increased lateral root numbers were also found in foxtail millet in our study. BR signaling mutants have been found to exhibit smaller leaf angles and more erect leaf habits than wild-type plants, whereas BR gain-of-function mutants show increased lamina joint bending [32]. These findings lay the foundation for further studies on BR signaling pathways in monocots.

The phosphorylation of BRI1, as an RLK, is very important for precise control of BR signals, and its phosphorylation sites have been reported in *Arabidopsis* and tomato [40, 41]. BRI1 comprises an extracellular domain, a TM domain, and a cytoplasmic domain [14]. The cytoplasmic domain contains the juxtamembrane region (JM), a serine/threonine/tyrosine kinase domain (KD), a the C-terminal (CT) domain. Most of the phosphorylation sites of AtBRI1 are located in the KD, such as Tyr-956, Thr-1039, Thr-1049, Ser-1044, and Thr-1045, and exhibit strong functions in BR signaling and plant growth [42–44]. Thr-1050 in the KD of tomato SlBRI1 negatively regulates the activity of SlBRI1 [40]. We also found 20 phosphorylation sites of OsBRI1 by in vitro kinase assay and mass spectrometry (Figure S2). Thr-963, Ser-966, Thr-969, Ser-1087 and Ser-1091 are conserved in *Arabidopsis* and rice. These phosphorylation sites were first found in OsBRI1, but their biological functions need further study. We also attempted to identify the phosphorylation sites of SiBRI1 in vivo by immunoprecipitation combined with mass spectrometry. Unfortunately, we did not find any post-translational modifications of SiBRI1 under BL treatment; this topic may warrant further study. We also found 128 SiBRI1 interaction proteins, we found SiBSK7, the homologous gene of AtBSKs, which helpful to analyze the components of BR signaling pathway in foxtail millet. In addition, RESISTANCE TO *PSEUDOMONAS SYRINGAE PV. MACULICOLA* 1 (SiRPM1) which response to defense [45], interacts with SiBRI1 through STING analysis [46], suggesting that SiBRI1 may be involved in disease resistance stress. To analyze the mechanism between SiBRI1 and interacting proteins is of great significance for elucidating the biological functions of SiBRI1 involved in multiple signaling pathways.

Overall, in this study, we identified the BR receptors in foxtail millet and divided the BRI1 family genes into three branches by phylogenetic analysis. Transformation of SiBRI1 restored the phenotype of *bri1-116*, and this complementation was a consequence of BR signaling pathway restoration. These results support SiBRI1 as a novel BRI1 gene in foxtail millet. Additionally, overexpression of SiBRI1 was found to modulate root development. Further investigations are needed to confirm the relationship between SiBRI1 and plant architecture, which may lead to the development of strategies to improve foxtail millet yield.

## Materials and methods

### BRI1 family gene identification and phylogenetic analysis

We identified foxtail millet SiBRI1 (Si000117m/XP_004969763), SiBRL1 (Si028727m/XP_004956489), SiBRL2 (Si013131m/XP_004983438) and SiBRL3 (Si033990m/XP_004973244) by blasting the AtBRI1 (AT4G39400) and OsBRI1 (LOC_Os01g52050) protein sequences in NCBI (https://www.ncbi.nlm.nih.gov/gene/), Phytozome 12 (https://phytozome.jgi.doe.gov/pz/portal.html#) and *Setaria italica* Functional Genomics Database (http://structuralbiology.cau.edu.cn/SIFGD/) websites with BLASTP algorithm. SiBRI family protein sequences, whole genomes and corresponding coding sequences (CDSs) were retrieved. Twenty-four representative plants with relatively complete annotated genome data were selected as the research subjects from the APG taxonomy [27] and phylogenetic relationship data. Taxonomic evolutionary relationships among species were visualized using the Timetree online tool (http://www.timetree.org/) [47, 48]. The genomic data were downloaded from the Ensembl Plants dataset (https://plants.ensembl.org) and the JGI plant database in Phytozome V12.1 (https://phytozome.jgi.doe.gov/pz/portal.html). For genome version information, see Table S1.

The HMM for the characteristic domain of BRI1 proteins was downloaded from the Pfam database (http://pfam.xfam.org) [49]. HMMER V 3.3 [28] was used to search for candidate genes with the whole protein sequences of each different species. Because the BRI1 family belongs to the RLK superfamily and because different members of the superfamily have similar domain compositions, it is difficult to search and identify BRI1 members in other species directly with the existing Pfam model. We analysed the results of the two multiple alignment methods (ClustalW and MUSCLE) and used three phylogenetic inference methods (NJ, maximum likelihood (ML), and minimum evolution (ME) in MEGA X [50] with 1000 bootstrap replicates to choose stable phylogenetic trees. Because of the need for functional divergence analysis and positive selection analysis, whole protein sequences were used to construct phylogenetic relationships among BRI1 gene family members. Among the plant species discussed, only angiosperms expressed the BRI1 gene in a strict sense.

### Plant growth and BL treatment

We received the *Arabidopsis* lines *bri1-116* from Zhiyong Wang (Carnegie Institution for Science, Stanford) [8], the *Arabidopsis* ecotype *Col-0* from the Arabidopsis Biological Resource Center (ABRC, www.arabidopsis.org). Yugu-1 received from Anyang District Institute of Agricultural Sciences in Henan province, Ci846 recieved from Crop Germplasm Resources in China (CGRIS, http://www.cgris.net/cgris_english.html). *Col-0* and the BR deletion mutant *bri1-116* were used in BRI1 overexpression and recovery experiments, and Ci846 was used in SiBRI1 overexpression experiments. *Arabidopsis* plants were grown in a chamber or incubator at 22 °C with a 16 h light/8 h dark (16L/8D) cycle, whereas foxtail millet plants were grown in a chamber or incubator at 28 °C with a 16L/8D cycle.

After the foxtail millet seeds were sterilized with chlorine gas (100 mL of 5% NaClO and 4 mL of HCl for 6–8 h), they were scattered on ^1^/_2_ MS medium (PhytoTechnology Laboratories, Overland Park, KS) with 1% sucrose and 0.3% Phytagel (PhytoTechnology Laboratories, Overland Park, KS), pH = 5.7. The plates were maintained under a 16L/8D cycle at 28 °C for 2 days. The seedlings with consistent growth were transferred to 6 × 20 cm (height) glass bottles containing one of several concentrations of BL in MS medium for further growth for 6 days before taking photos, removed for root length and lateral root number measurement, or cleared for an Asana microscopic observation. The lengths of the primary roots were measured using Image J software. 8-day-old *pUbi:SiBRI1-eGFP/Ci846* seedlings were immersed in 1 μM BL for 1 h, and leaves and roots were placed into liquid nitrogen immediately after treatment. Then, RNA was extracted to detect BR synthesis gene expression.

After the *Arabidopsis* seeds were sterilized with 75% ethanol and 0.1% Triton X-100 for 5 min, they were grown on ^1^/_2_ MS medium with 1% sucrose and 0.45% Phytagel containing different concentrations of BL and PCZ. The plates were maintained under a 16L/8D cycle at 22 °C for 7 days. Photographs were taken, and the lengths of the primary roots were measured using Image J software. For clearly present the measurement data, we shown the relative length. We set the lengths of the primary roots under 0 nM BL or hypocotyls under 0 μM PCZ as 1, and the 5–100 nM BL or 0.25 μM PCZ group calculated the relative value respectively.

### qRT-PCR

Total RNA was extracted according to Zhao (2021) [31]. First-strand cDNA was synthesized from approximately 1 µg of total RNA using M-MLV Reverse Transcriptase (Takara Bio, Inc., Otsu, Japan). qRT-PCR was performed according to a standard protocol using a Bio-Rad CFX Connect Real-Time PCR machine (Bio-Rad Laboratories, Hercules, CA, United States) and the SYBR Premix Ex Taq™ system (Takara Bio, Inc.). The primers used are listed in Table S6, and *SiActin* was used as an internal reference. The average value from at least three biological replicates is presented.

### Subcellular localization of SiBRI1

pUbi:SiBRI1-eGFP was constructed by using a recombinant cloning technique. First, the SiBRI1 CDS was cloned with a pCR™8/GW/TOPO® TA Cloning Kit (Invitrogen) and then cloned into the modified binary vector pCAMBIA1305-eGFP via an LR enzyme (Invitrogen). pCAMBIA1305-eGFP contains a maize ubiquitin promoter and a C-terminal enhanced GFP tag. The expression vectors were incorporated into *Agrobacterium tumefaciens* strain EHA105 and transformed into Ci846 with callus-based gene transformation procedures.

The pUbi:SiBRI1-eGFP transgenic plants were grown under a 16L/8D cycle at 28 °C on ^1^/_2_ MS medium for 3 days. The root tips were collected, and an FV3000 confocal microscope was used to stimulate GFP signals at a wavelength of 488 nm. The emission signals were collected and transmitted at a wavelength of 518 nm.

### Immunoblotting

One-week-old and two-week-old *Arabidopsis* seedlings and foxtail millet leaves were ground to a fine powder in liquid nitrogen. Immunoblotting was then performed using anti-GFP (*ProteinFind*® Anti-GFP Mouse Monoclonal Antibody, TRANS, Beijing, China) and anti-BZR1 (Cat: YKZPK82, Youke Biotechnology, Shanghai, China) antibodies.

### Genotype identification of *bri1-116* plants

After three weeks of growth of *bri1-116* plants, DNA was extracted. The PCR primers BRI1-seq3 (CCAAATCTCTgCCAgAACCC) and BRI1-116GT-R (TACCTCATCAGGAATCGAACCAG), which produce an amplicon 852 bp in size, were used. The *Col-0* product was cut into two bands (347 bp and 505 bp) by the *MssI* enzyme. The *bri1-116* product could not be cut by the *MssI* enzyme due to point mutation; thus, the product remained 852 bp in size.

### Immunoprecipitation and LC–MS/MS identification

Ci846 seeds were germinated and then grown in double-distilled water at 28 °C under LD conditions for 4 days. Seedlings with similar root and coleoptile lengths were selected and transferred to ^1^/_2_ strength Hoagland solution, and allowed to grow for 4 more days. The leaves were then immersed in ^1^/_2_ strength Hoagland solution with 1 μM BL or not for 2 h, and leaves were placed into liquid nitrogen immediately after treatment and ground to a fine powder in liquid nitrogen. The tissues were extracted using a Grinding buffer containing 25 mM HEPES, 0.6% PVP, 5 mM VC, 5 mM EDTA, 25 mM NaF, 1 mM Na_2_MoO_4_, 1 mM Na_3_VO_4_, 5 mM DTT, 2 mM imidazole and protease inhibitor cocktails (Sigma) at 4 °C, and centrifuged at 4 °C for 10 min and 2000 × g. The homogenate was centrifuged at 10,000 g for 45 min and the deposit was extracted using a NEB buffer containing 20 mM HEPES (pH 7.5), 40 mM KCl, 1 mM EDTA, 1% TritonX-100 and protease inhibitor, then incubated with GFP-Trap Agarose (Ychromotek, gta) for 2 h at 4 °C. The Beads were washed on a column with 20 bed volumes of NEB buffer and then eluted with SDS loading buffer (0.125 M Tris–HCl (pH 6.8), 2% β-Mercaptoethanol, 4% SDS, 20% glycerol, 0.25% bromophenol blue). Proteins eluted from the GFP-Trap were separated by 8–20% Precast-GLgel Tris–Glycine (BBI-Rad, E919DA0304), and each line was cut into four pieces according to the molecular weight of the proteins. Protein in-gel digestion and LC–MS/MS identification was performed according to our published method [51, 52].

## Supplementary Information

**Additional file 1: Figure S1.** A model shows BR signaling pathways. BRs are recognized by BR receptor BRI1 and its coreceptor BAK1. BR promotes the association of BRI1 with BAK1 and enables transphosphorylation between the cytoplasmic kinase domains of the two receptors. BRI1 then phosphorylates BSKs and CDG1, leading to activation of BSU1. BSU1 dephosphorylates and inhibits BIN2. In the absence of BRs, BIN2 phosphorylates BZRs family, preventing them from regulating the transcription of downstream target genes. BR signaling inhibits the kinase activity of BIN2 and allows BZRs to be dephosphorylated by PP2A. Dephosphorylated BZRs bind to BR response elements (BRRE) or E-box cis-elements and regulate the expression of many BR-responsive genes. **Figure S2.** Taxonomic relationships among 28 representative plants. **Figure S3.**. Characterization of the SiBRI1 protein. AtBRI1, OsBRI1 and SiBRI1 protein sequences were downloaded from the Phytozome 12 website (https://phytozome.jgi.doe.gov/pz/portal.html#) using the multiple sequence analysis web tool ClustalW (https://www.genome.jp/tools-bin/clustalw). The black lines indicate a conserved signal peptide, a putative Leu zipper motif, two conservatively spaced cysteine pairs and a predicted TM domain. The black box indicates 12 conserved protein kinase domains (labelled I to XI), and the red letters indicate phosphorylation sites in *Arabidopsis* and rice. **Figure S4.** SiBRI1 overexpression activated BR signaling in *Arabidopsis*. (A), The phenotype of *SiBRI1-OX/Col* which was grown in the presence of indicated concentration of PCZ for 7 days. bar=1cm. (B), Relative hypocotyl length (A) was quantified. Error bars indicate the mean ± standard deviation (SD). Statistically significant differences are indicated by different lowercase letters (*p*<0.05, two-way ANOVA with Tukey’s significant difference test). (C), Expression levels of SiBRI1-YFP and AtBZR1 in the transgenic plants shown in (A). Ponceau S staining of the Rubisco large subunit and the expression of HSP70 was used as an equal loading control. (D), Phenotype of light-grown 4-week-old *SiBRI1-OX/Col.* **Figure S5.** The expression level of *SiBR11* under BL treament. The expression level of *SiBRI1* in leaves and roots under 1μM BL immersed for 1 hours. Three biological repetitions. Error bars indicate the mean ± standard deviation (SD). Statistically significant differences are indicated by different lowercase letters (*p*<0.05, one-way ANOVA with Tukey’s significant difference test). **Figure S6.** The expression level of *SiBR11/Ci846*. The expression level of SiBRI1 in overexpression line in Figure 5C, Ponceau S staining of the Rubisco large subunit was used as an equal loading control.

**Additional file 2.**

**Additional file 3.**

## Data Availability

The protein sequences and amino acid sequences information of foxtail millet, rice and Arabidopsis BRI1 gene were collected from Setaria italica Functional Genomics Database (http://structuralbiology.cau.edu.cn/SIFGD/), NCBI (https://www.ncbi.nlm.nih.gov/gene/) and The Arabidopsis Information Resource (https://www.arabidopsis.org/), respectively. The genomic data of twenty-four representative plants of BRI1 family genes were downloaded from the Ensembl Plants dataset (https://plants.ensembl.org) and the JGI plant database in Phytozome V12.1 (https://phytozome.jgi.doe.gov/pz/portal.html). Venn diagram of the proteins was performed online using InteractiVenn (http://www.interactivenn.net). Gene Ontology (GO) annotation and enrichment analysis was performed online using AgriGO (http://bioinfo.cau.edu.cn/agriGO/). All data generated or analyzed during this study are included in this published article and its supplementary information files. The plant materials and recombinant plasmids generated during this study are available from the corresponding author on reasonable request.

## Abbreviations

BRs: Brassinosteroids
BL: Brassinolide
PCZ: Propiconazole
RLCKs: RECEPTOR-LIKE CYTOPLASMIC KINASEs
BRI1: BRASSINOSTEROID INSENSITIVE 1
BRLs: BRI1-LIKE RECEPTOR KINASEs
BAK1: BRI1-ASSOCIATED RECEPTOR KINASE 1
BSKs: BR SIGNALING KINASEs
CDG1: CONSTITUTIVE DIFFERENTIAL GROWTH 1
BSU1: BRI1 SUPPRESSOR 1
BIN2: BR INSENSITIVE 2
PP2A: PROTEIN PHOSPHATASE 2A
BZRs: BRASSINAZOLE RESISTANTs
DPY1: DROOPY LEAF1
CPD: CONSTITUTIVE PHOTOMORPHOGENIC DWARF
DWF4: DWARF 4
GSK3: GLYCOGEN SYNTHASE KINASE 3
PLT1: PLETHORA 1
LBD16: LATERAL ORGAN BOUNDARIES DOMAIN16
qRT-PCR: Quantitative real-time PCR
IP: Immunoprecipitation
MS: Mass Spectrometry
LC–MS/MS: Liquid chromatography tandem mass spectrometry
APG: Angiosperm Phylogeny Group
HMM: Hidden Markov model
*bri1-5*, *bri1-116*: BR-insensitive mutant
