# Supplementary material for: Evolutionary analysis and functional characterization of SiBRI1 as a Brassinosteroid receptor gene in foxtail millet
Source: BMC Plant Biol. 2021 Jun 24;21:291. doi: 10.1186/s12870-021-03081-8 (PMC8223282; doi:10.1186/s12870-021-03081-8)
Supplement: Supplementary file 1 — Additional file 1: Figure S1. A model shows BR signaling pathways. BRs are recognized by BR receptor BRI1 and its coreceptor BAK1. BR promotes the association of BRI1 with BAK1 and enables transphosphorylation between the cytoplasmic kinase domains of the two receptors. BRI1 then phosphorylates BSKs and CDG1, leading to activation of BSU1. BSU1 dephosphorylates and inhibits BIN2. In the absence of BRs, BIN2 phosphorylates BZRs family, preventing them from regulating the transcription of downstream target genes. BR signaling inhibits the kinase activity of BIN2 and allows BZRs to be dephosphorylated by PP2A. Dephosphorylated BZRs bind to BR response elements (BRRE) or E-box cis-elements and regulate the expression of many BR-responsive genes. Figure S2. Taxonomic relationships among 28 representative plants. Figure S3.. Characterization of the SiBRI1 protein. AtBRI1, OsBRI1 and SiBRI1 protein sequences were downloaded from the Phytozome 12 website (https://phytozome.jgi.doe.gov/pz/portal.html#) using the multiple sequence analysis web tool ClustalW (https://www.genome.jp/tools-bin/clustalw). The black lines indicate a conserved signal peptide, a putative Leu zipper motif, two conservatively spaced cysteine pairs and a predicted TM domain. The black box indicates 12 conserved protein kinase domains (labelled I to XI), and the red letters indicate phosphorylation sites in Arabidopsis and rice. Figure S4. SiBRI1 overexpression activated BR signaling in Arabidopsis. (A), The phenotype of SiBRI1-OX/Col which was grown in the presence of indicated concentration of PCZ for 7 days. bar=1cm. (B), Relative hypocotyl length (A) was quantified. Error bars indicate the mean ± standard deviation (SD). Statistically significant differences are indicated by different lowercase letters (p<0.05, two-way ANOVA with Tukey’s significant difference test). (C), Expression levels of SiBRI1-YFP and AtBZR1 in the transgenic plants shown in (A). Ponceau S staining of the Rubisco large subunit [file 12870_2021_3081_MOESM1_ESM.pptx]

## Slide 1
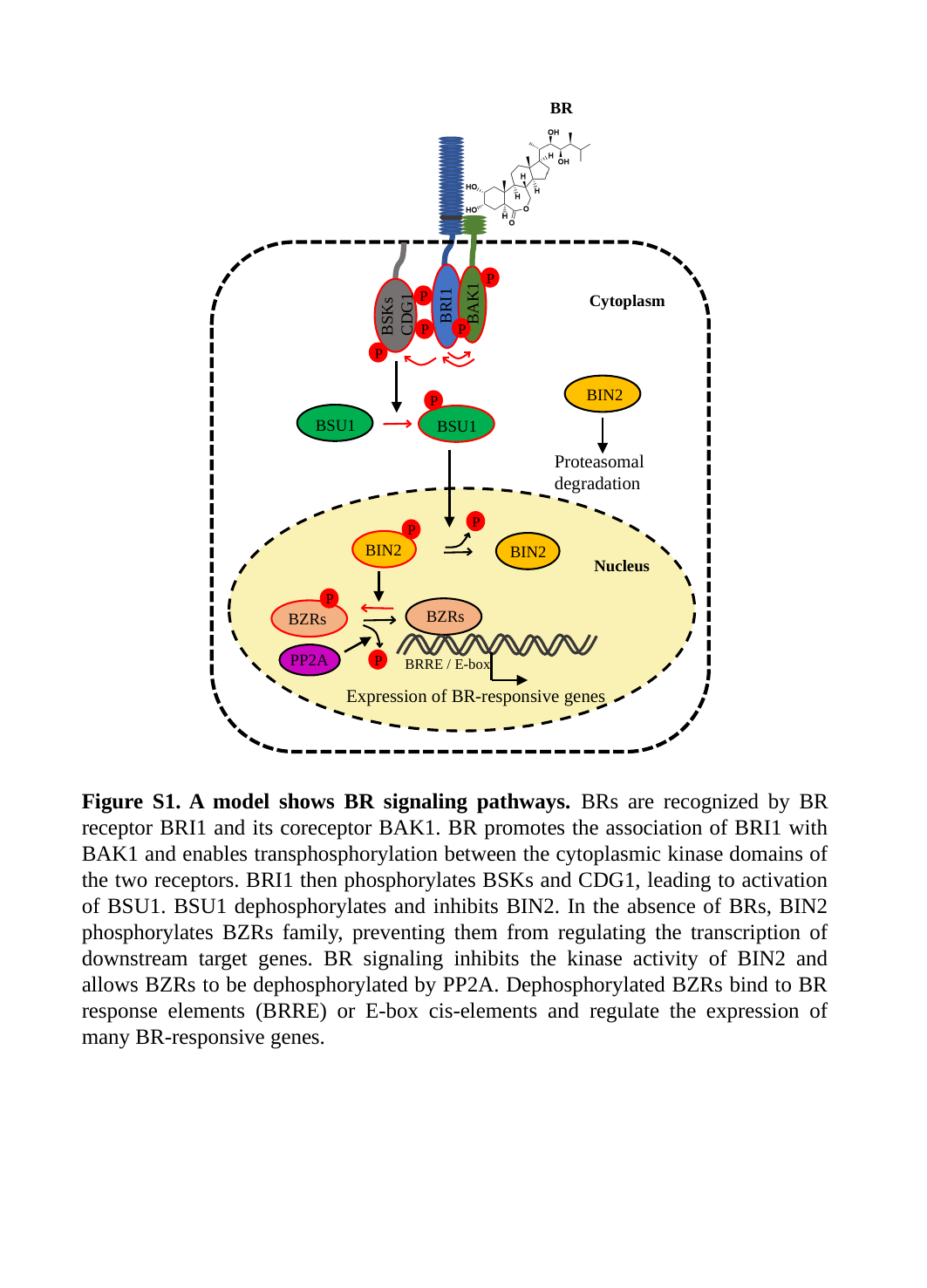

BR
P
P
BSKs
CDG1
Cytoplasm
BAK1
BRI1
P
P
P
BIN2
P
BSU1
BSU1
Proteasomal
degradation
P
P
BIN2
BIN2
Nucleus
P
BZRs
BZRs
PP2A
P
BRRE / E-box
Expression of BR-responsive genes
Figure S1. A model shows BR signaling pathways. BRs are recognized by BR receptor BRI1 and its coreceptor BAK1. BR promotes the association of BRI1 with BAK1 and enables transphosphorylation between the cytoplasmic kinase domains of the two receptors. BRI1 then phosphorylates BSKs and CDG1, leading to activation of BSU1. BSU1 dephosphorylates and inhibits BIN2. In the absence of BRs, BIN2 phosphorylates BZRs family, preventing them from regulating the transcription of downstream target genes. BR signaling inhibits the kinase activity of BIN2 and allows BZRs to be dephosphorylated by PP2A. Dephosphorylated BZRs bind to BR response elements (BRRE) or E-box cis-elements and regulate the expression of many BR-responsive genes.

## Slide 2
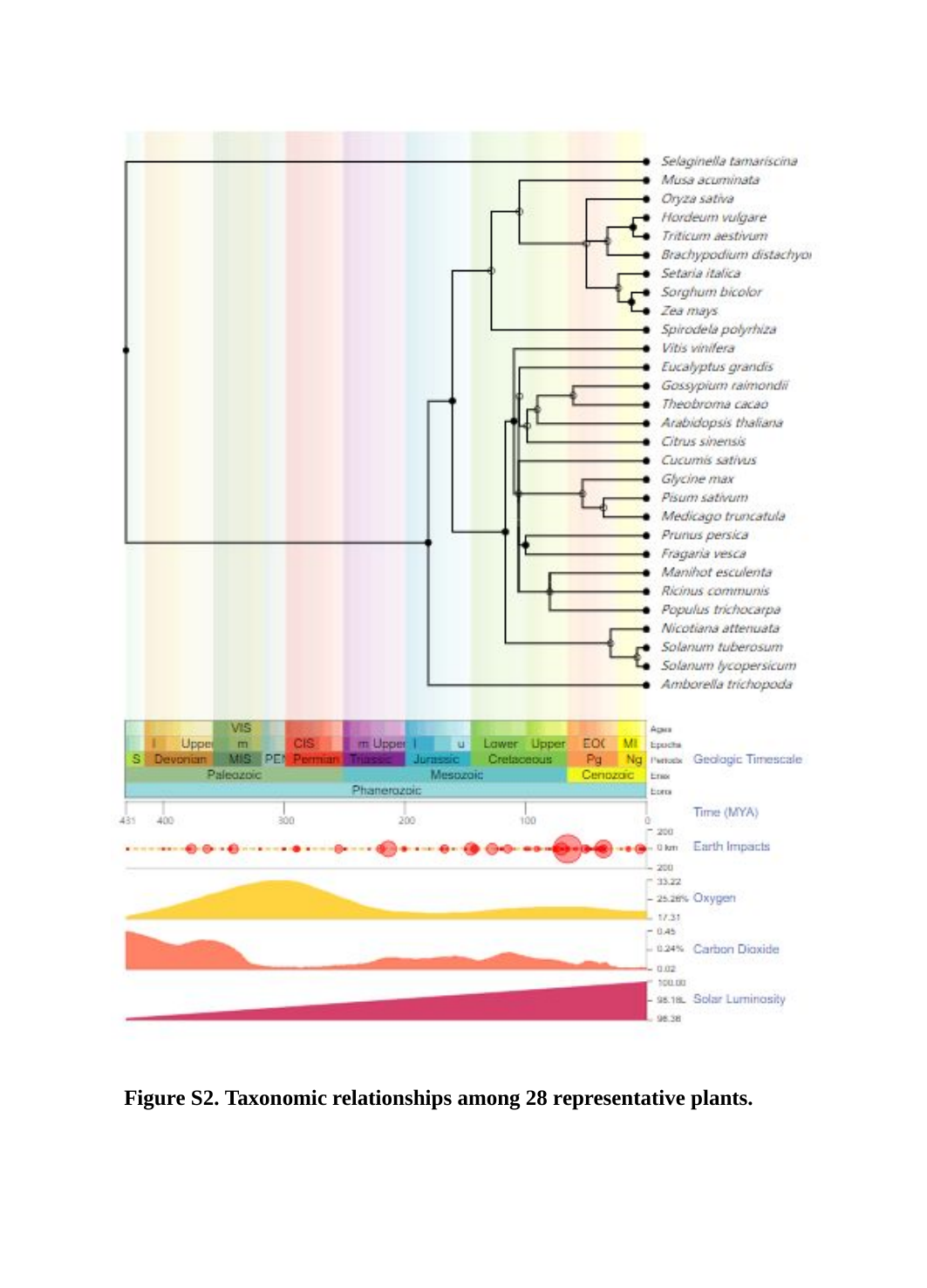

Figure S2. Taxonomic relationships among 28 representative plants.

## Slide 3
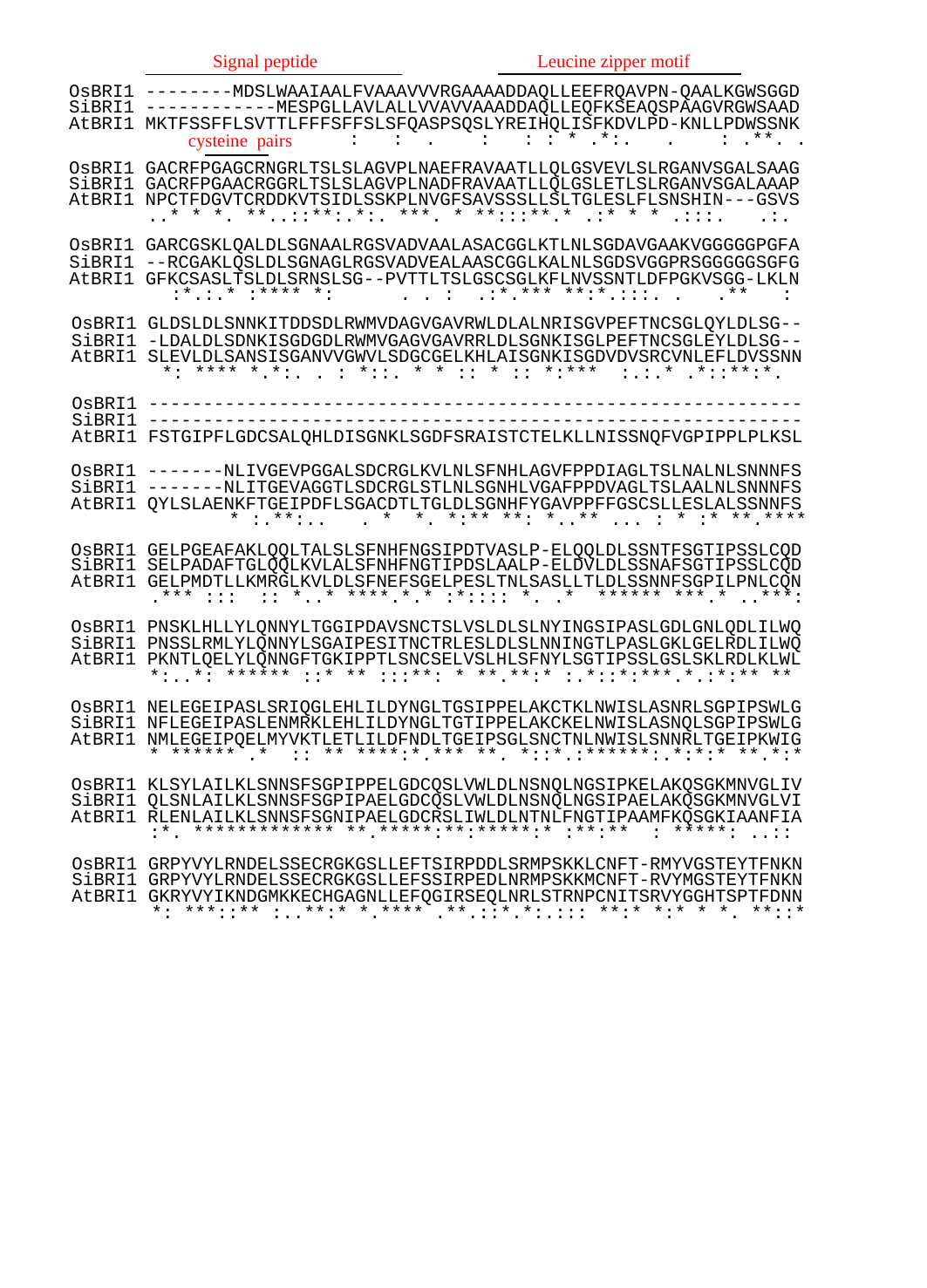

Signal peptide
Leucine zipper motif
OsBRI1 --------MDSLWAAIAALFVAAAVVVRGAAAADDAQLLEEFRQAVPN-QAALKGWSGGD
SiBRI1 ------------MESPGLLAVLALLVVAVVAAADDAQLLEQFKSEAQSPAAGVRGWSAAD
AtBRI1 MKTFSSFFLSVTTLFFFSFFSLSFQASPSQSLYREIHQLISFKDVLPD-KNLLPDWSSNK
: : . : : : * .*:. . : .**. .
cysteine pairs
OsBRI1 GACRFPGAGCRNGRLTSLSLAGVPLNAEFRAVAATLLQLGSVEVLSLRGANVSGALSAAG
SiBRI1 GACRFPGAACRGGRLTSLSLAGVPLNADFRAVAATLLQLGSLETLSLRGANVSGALAAAP
AtBRI1 NPCTFDGVTCRDDKVTSIDLSSKPLNVGFSAVSSSLLSLTGLESLFLSNSHIN---GSVS
..* * *. **..::**:.*:. ***. * **:::**.* .:* * * .:::. .:.
OsBRI1 GARCGSKLQALDLSGNAALRGSVADVAALASACGGLKTLNLSGDAVGAAKVGGGGGPGFA
SiBRI1 --RCGAKLQSLDLSGNAGLRGSVADVEALAASCGGLKALNLSGDSVGGPRSGGGGGSGFG
AtBRI1 GFKCSASLTSLDLSRNSLSG--PVTTLTSLGSCSGLKFLNVSSNTLDFPGKVSGG-LKLN
:*.:.* :**** *: . . : .:*.*** **:*.:::. . .** :
OsBRI1 GLDSLDLSNNKITDDSDLRWMVDAGVGAVRWLDLALNRISGVPEFTNCSGLQYLDLSG--
SiBRI1 -LDALDLSDNKISGDGDLRWMVGAGVGAVRRLDLSGNKISGLPEFTNCSGLEYLDLSG--
AtBRI1 SLEVLDLSANSISGANVVGWVLSDGCGELKHLAISGNKISGDVDVSRCVNLEFLDVSSNN
*: **** *.*:. . : *::. * * :: * :: *:*** :.:.* .*::**:*.
OsBRI1 ------------------------------------------------------------
SiBRI1 ------------------------------------------------------------
AtBRI1 FSTGIPFLGDCSALQHLDISGNKLSGDFSRAISTCTELKLLNISSNQFVGPIPPLPLKSL
OsBRI1 -------NLIVGEVPGGALSDCRGLKVLNLSFNHLAGVFPPDIAGLTSLNALNLSNNNFS
SiBRI1 -------NLITGEVAGGTLSDCRGLSTLNLSGNHLVGAFPPDVAGLTSLAALNLSNNNFS
AtBRI1 QYLSLAENKFTGEIPDFLSGACDTLTGLDLSGNHFYGAVPPFFGSCSLLESLALSSNNFS
* :.**:.. . * *. *:** **: *..** ... : * :* **.****
OsBRI1 GELPGEAFAKLQQLTALSLSFNHFNGSIPDTVASLP-ELQQLDLSSNTFSGTIPSSLCQD
SiBRI1 SELPADAFTGLQQLKVLALSFNHFNGTIPDSLAALP-ELDVLDLSSNAFSGTIPSSLCQD
AtBRI1 GELPMDTLLKMRGLKVLDLSFNEFSGELPESLTNLSASLLTLDLSSNNFSGPILPNLCQN
.*** ::: :: *..* ****.*.* :*:::: *. .* ****** ***.* ..***:
OsBRI1 PNSKLHLLYLQNNYLTGGIPDAVSNCTSLVSLDLSLNYINGSIPASLGDLGNLQDLILWQ
SiBRI1 PNSSLRMLYLQNNYLSGAIPESITNCTRLESLDLSLNNINGTLPASLGKLGELRDLILWQ
AtBRI1 PKNTLQELYLQNNGFTGKIPPTLSNCSELVSLHLSFNYLSGTIPSSLGSLSKLRDLKLWL
*:..*: ****** ::* ** :::**: * **.**:* :.*::*:***.*.:*:** **
OsBRI1 NELEGEIPASLSRIQGLEHLILDYNGLTGSIPPELAKCTKLNWISLASNRLSGPIPSWLG
SiBRI1 NFLEGEIPASLENMRKLEHLILDYNGLTGTIPPELAKCKELNWISLASNQLSGPIPSWLG
AtBRI1 NMLEGEIPQELMYVKTLETLILDFNDLTGEIPSGLSNCTNLNWISLSNNRLTGEIPKWIG
* ****** .* :: ** ****:*.*** **. *::*.:******:.*:*:* **.*:*
OsBRI1 KLSYLAILKLSNNSFSGPIPPELGDCQSLVWLDLNSNQLNGSIPKELAKQSGKMNVGLIV
SiBRI1 QLSNLAILKLSNNSFSGPIPAELGDCQSLVWLDLNSNQLNGSIPAELAKQSGKMNVGLVI
AtBRI1 RLENLAILKLSNNSFSGNIPAELGDCRSLIWLDLNTNLFNGTIPAAMFKQSGKIAANFIA
:*. ************* **.*****:**:*****:* :**:** : *****: ..::
OsBRI1 GRPYVYLRNDELSSECRGKGSLLEFTSIRPDDLSRMPSKKLCNFT-RMYVGSTEYTFNKN
SiBRI1 GRPYVYLRNDELSSECRGKGSLLEFSSIRPEDLNRMPSKKMCNFT-RVYMGSTEYTFNKN
AtBRI1 GKRYVYIKNDGMKKECHGAGNLLEFQGIRSEQLNRLSTRNPCNITSRVYGGHTSPTFDNN
*: ***::** :..**:* *.**** .**.::*.*:.::: **:* *:* * *. **::*

## Slide 4
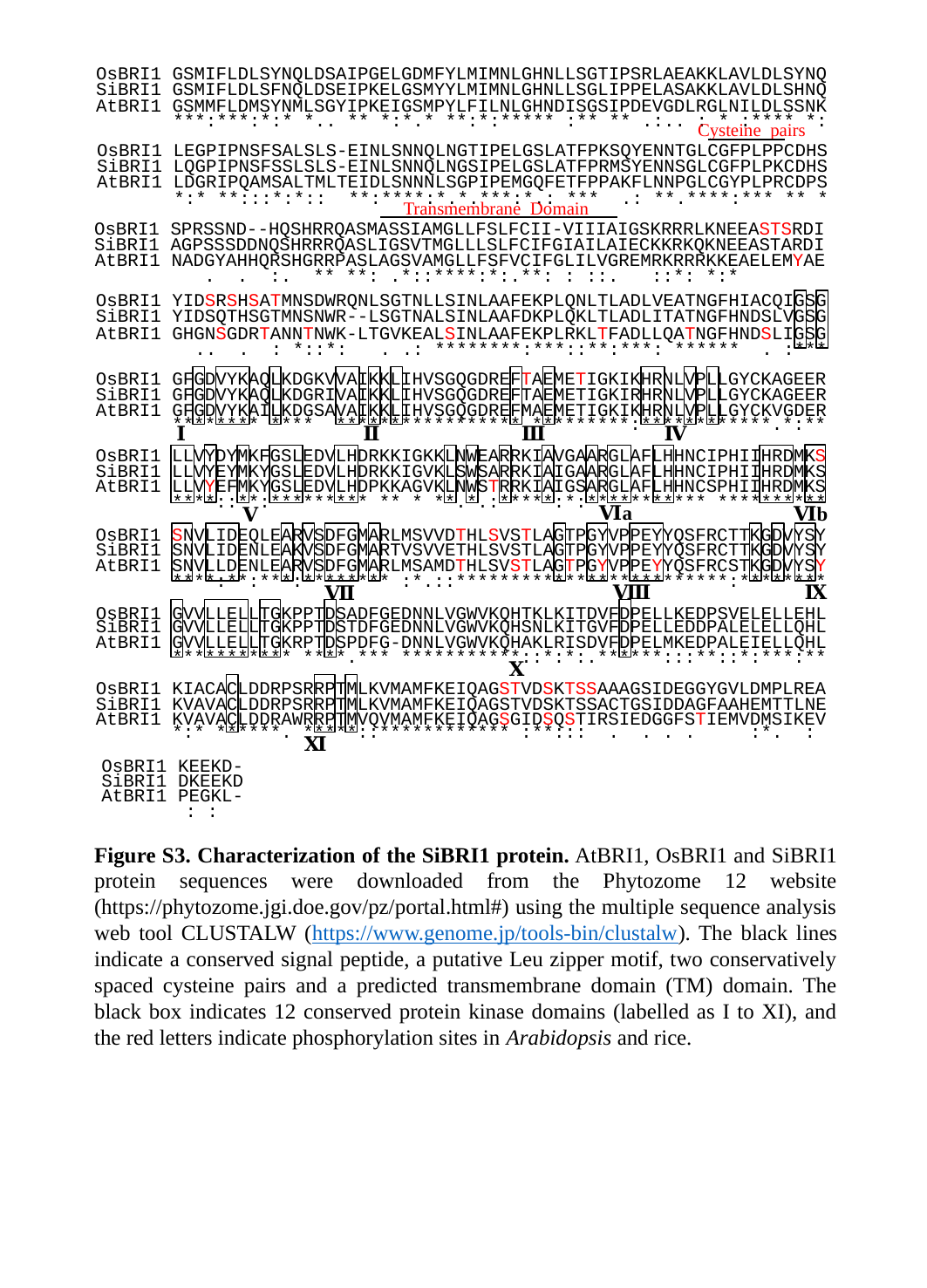

OsBRI1 GSMIFLDLSYNQLDSAIPGELGDMFYLMIMNLGHNLLSGTIPSRLAEAKKLAVLDLSYNQ
SiBRI1 GSMIFLDLSFNQLDSEIPKELGSMYYLMIMNLGHNLLSGLIPPELASAKKLAVLDLSHNQ
AtBRI1 GSMMFLDMSYNMLSGYIPKEIGSMPYLFILNLGHNDISGSIPDEVGDLRGLNILDLSSNK
***:***:*:* *.. ** *:*.* **:*:***** :** ** .:.. : * :**** *:
Cysteine pairs
OsBRI1 LEGPIPNSFSALSLS-EINLSNNQLNGTIPELGSLATFPKSQYENNTGLCGFPLPPCDHS
SiBRI1 LQGPIPNSFSSLSLS-EINLSNNQLNGSIPELGSLATFPRMSYENNSGLCGFPLPKCDHS
AtBRI1 LDGRIPQAMSALTMLTEIDLSNNNLSGPIPEMGQFETFPPAKFLNNPGLCGYPLPRCDPS
*:* **:::*:*:: **:****:*.*.***:*.: *** .: **.****:*** ** *
Transmembrane Domain
OsBRI1 SPRSSND--HQSHRRQASMASSIAMGLLFSLFCII-VIIIAIGSKRRRLKNEEASTSRDI
SiBRI1 AGPSSSDDNQSHRRRQASLIGSVTMGLLLSLFCIFGIAILAIECKKRKQKNEEASTARDI
AtBRI1 NADGYAHHQRSHGRRPASLAGSVAMGLLFSFVCIFGLILVGREMRKRRRKKEAELEMYAE
. . :. ** **: .*::****:*:.**: : ::. ::*: *:*
OsBRI1 YIDSRSHSATMNSDWRQNLSGTNLLSINLAAFEKPLQNLTLADLVEATNGFHIACQIGSG
SiBRI1 YIDSQTHSGTMNSNWR--LSGTNALSINLAAFDKPLQKLTLADLITATNGFHNDSLVGSG
AtBRI1 GHGNSGDRTANNTNWK-LTGVKEALSINLAAFEKPLRKLTFADLLQATNGFHNDSLIGSG
.. . : *::*: . .: ********:***::**:***: ****** . :***
OsBRI1 GFGDVYKAQLKDGKVVAIKKLIHVSGQGDREFTAEMETIGKIKHRNLVPLLGYCKAGEER
SiBRI1 GFGDVYKAQLKDGRIVAIKKLIHVSGQGDREFTAEMETIGKIRHRNLVPLLGYCKAGEER
AtBRI1 GFGDVYKAILKDGSAVAIKKLIHVSGQGDREFMAEMETIGKIKHRNLVPLLGYCKVGDER
******** **** ***************** *********:************.*:**
Ⅰ
Ⅱ
Ⅲ
Ⅳ
OsBRI1 LLVYDYMKFGSLEDVLHDRKKIGKKLNWEARRKIAVGAARGLAFLHHNCIPHIIHRDMKS
SiBRI1 LLVYEYMKYGSLEDVLHDRKKIGVKLSWSARRKIAIGAARGLAFLHHNCIPHIIHRDMKS
AtBRI1 LLVYEFMKYGSLEDVLHDPKKAGVKLNWSTRRKIAIGSARGLAFLHHNCSPHIIHRDMKS
****::**:********* ** * **.*.:*****:*:*********** **********
Ⅵa
Ⅵb
Ⅴ
OsBRI1 SNVLIDEQLEARVSDFGMARLMSVVDTHLSVSTLAGTPGYVPPEYYQSFRCTTKGDVYSY
SiBRI1 SNVLIDENLEAKVSDFGMARTVSVVETHLSVSTLAGTPGYVPPEYYQSFRCTTKGDVYSY
AtBRI1 SNVLLDENLEARVSDFGMARLMSAMDTHLSVSTLAGTPGYVPPEYYQSFRCSTKGDVYSY
****:**:***:******** :*.::*************************:********
Ⅷ
Ⅸ
Ⅶ
OsBRI1 GVVLLELLTGKPPTDSADFGEDNNLVGWVKQHTKLKITDVFDPELLKEDPSVELELLEHL
SiBRI1 GVVLLELLTGKPPTDSTDFGEDNNLVGWVKQHSNLKITGVFDPELLEDDPALELELLQHL
AtBRI1 GVVLLELLTGKRPTDSPDFG-DNNLVGWVKQHAKLRISDVFDPELMKEDPALEIELLQHL
*********** ****.*** ***********::*:*:.******:::**::*:***:**
Ⅹ
OsBRI1 KIACACLDDRPSRRPTMLKVMAMFKEIQAGSTVDSKTSSAAAGSIDEGGYGVLDMPLREA
SiBRI1 KVAVACLDDRPSRRPTMLKVMAMFKEIQAGSTVDSKTSSACTGSIDDAGFAAHEMTTLNE
AtBRI1 KVAVACLDDRAWRRPTMVQVMAMFKEIQAGSGIDSQSTIRSIEDGGFSTIEMVDMSIKEV
*:* ******. *****::************ :**::: . . . . :*. :
Ⅺ
OsBRI1 KEEKD-
SiBRI1 DKEEKD
AtBRI1 PEGKL-
: :
Figure S3. Characterization of the SiBRI1 protein. AtBRI1, OsBRI1 and SiBRI1 protein sequences were downloaded from the Phytozome 12 website (https://phytozome.jgi.doe.gov/pz/portal.html#) using the multiple sequence analysis web tool CLUSTALW (https://www.genome.jp/tools-bin/clustalw). The black lines indicate a conserved signal peptide, a putative Leu zipper motif, two conservatively spaced cysteine pairs and a predicted transmembrane domain (TM) domain. The black box indicates 12 conserved protein kinase domains (labelled as I to XI), and the red letters indicate phosphorylation sites in Arabidopsis and rice.

## Slide 5
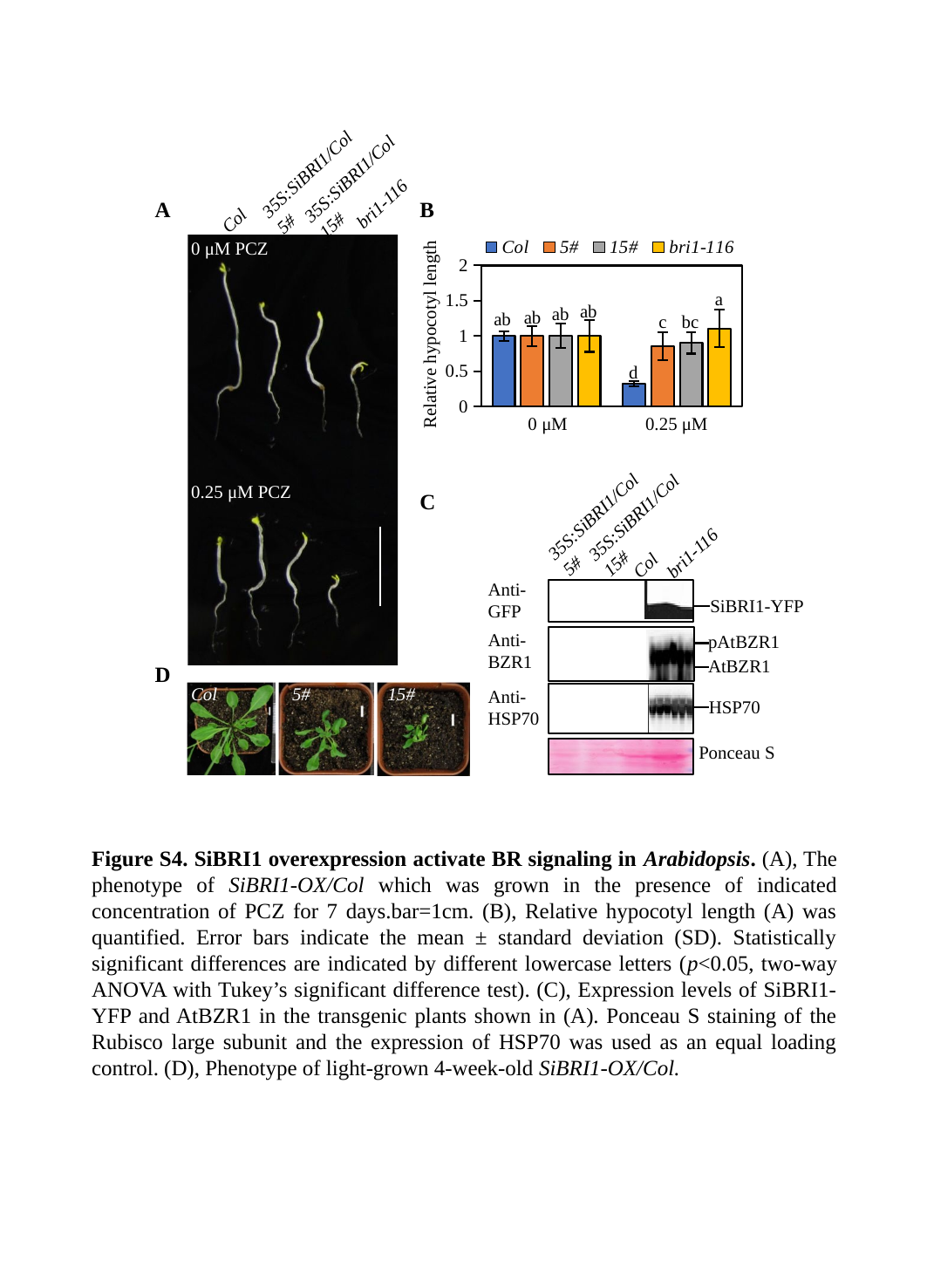

35S:SiBRI1/Col
5#
35S:SiBRI1/Col
15#
bri1-116
Col
A
B
### Chart
| Category | Col | 5# | 15# | bri1-116 |
|---|---|---|---|---|
| 0μM | 0.999999999999997 | 1.0000000000000002 | 0.9999999999999997 | 1.0 |
| 0.25μM | 0.32324506833683536 | 0.8578582236835999 | 0.9009540884363477 | 1.1054815862168283 |0 μM PCZ
a
ab
ab
ab
ab
c
bc
Relative hypocotyl length
d
0 μM
0.25 μM
0.25 μM PCZ
35S:SiBRI1/Col
5#
C
35S:SiBRI1/Col
15#
Col
bri1-116
Anti-GFP
SiBRI1-YFP
Anti-BZR1
pAtBZR1
AtBZR1
D
15#
Col
5#
Anti-HSP70
HSP70
Ponceau S
Figure S4. SiBRI1 overexpression activate BR signaling in Arabidopsis. (A), The phenotype of SiBRI1-OX/Col which was grown in the presence of indicated concentration of PCZ for 7 days.bar=1cm. (B), Relative hypocotyl length (A) was quantified. Error bars indicate the mean ± standard deviation (SD). Statistically significant differences are indicated by different lowercase letters (p<0.05, two-way ANOVA with Tukey’s significant difference test). (C), Expression levels of SiBRI1-YFP and AtBZR1 in the transgenic plants shown in (A). Ponceau S staining of the Rubisco large subunit and the expression of HSP70 was used as an equal loading control. (D), Phenotype of light-grown 4-week-old SiBRI1-OX/Col.

## Slide 6
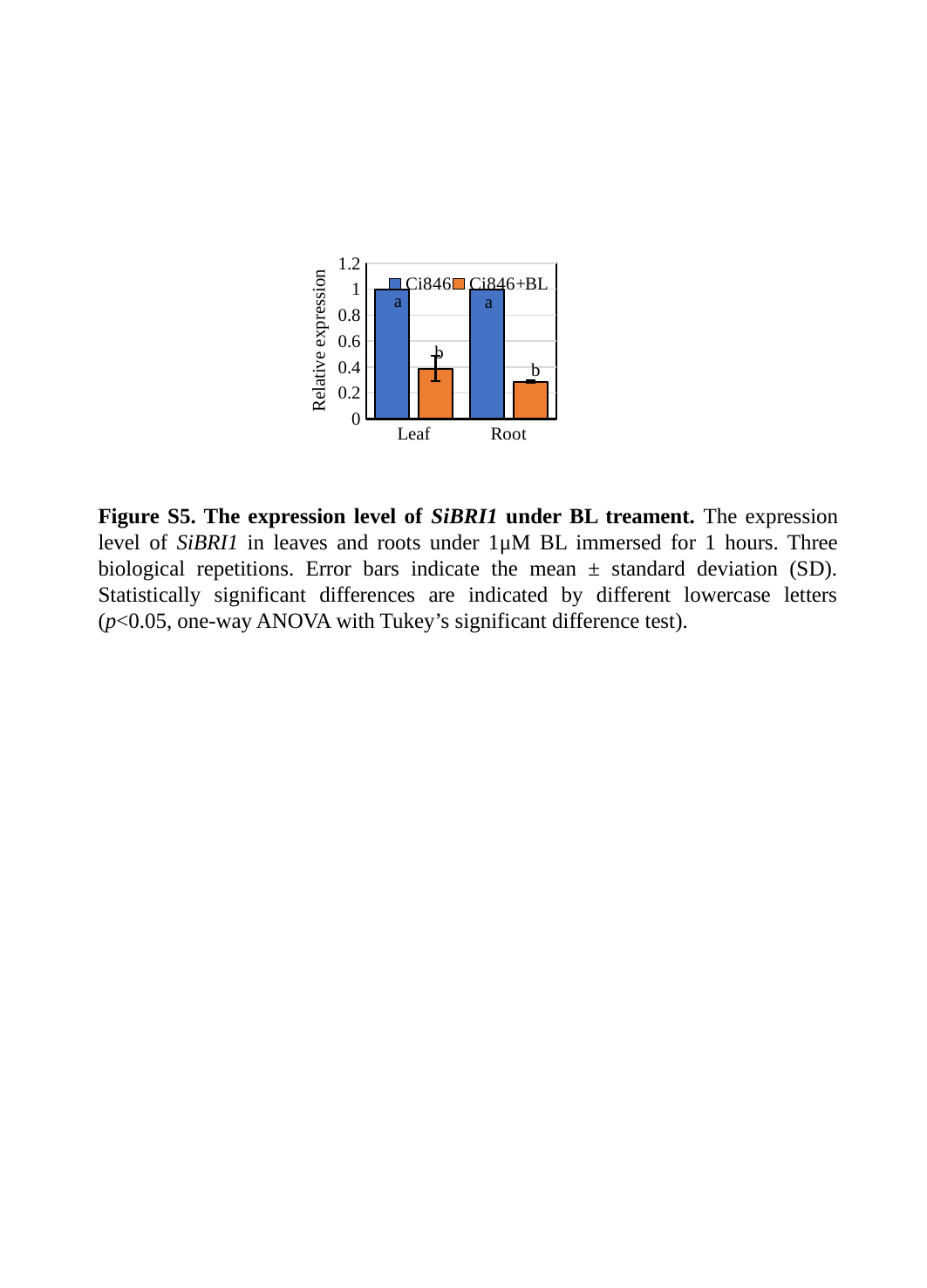

### Chart
| Category | Ci846 | Ci846+BL |
|---|---|---|
| Leaf | 1.0 | 0.38843 |
| Root | 1.0 | 0.28768666666666665 |a
a
Relative expression
b
b
Figure S5. The expression level of SiBRI1 under BL treament. The expression level of SiBRI1 in leaves and roots under 1μM BL immersed for 1 hours. Three biological repetitions. Error bars indicate the mean ± standard deviation (SD). Statistically significant differences are indicated by different lowercase letters (p<0.05, one-way ANOVA with Tukey’s significant difference test).

## Slide 7
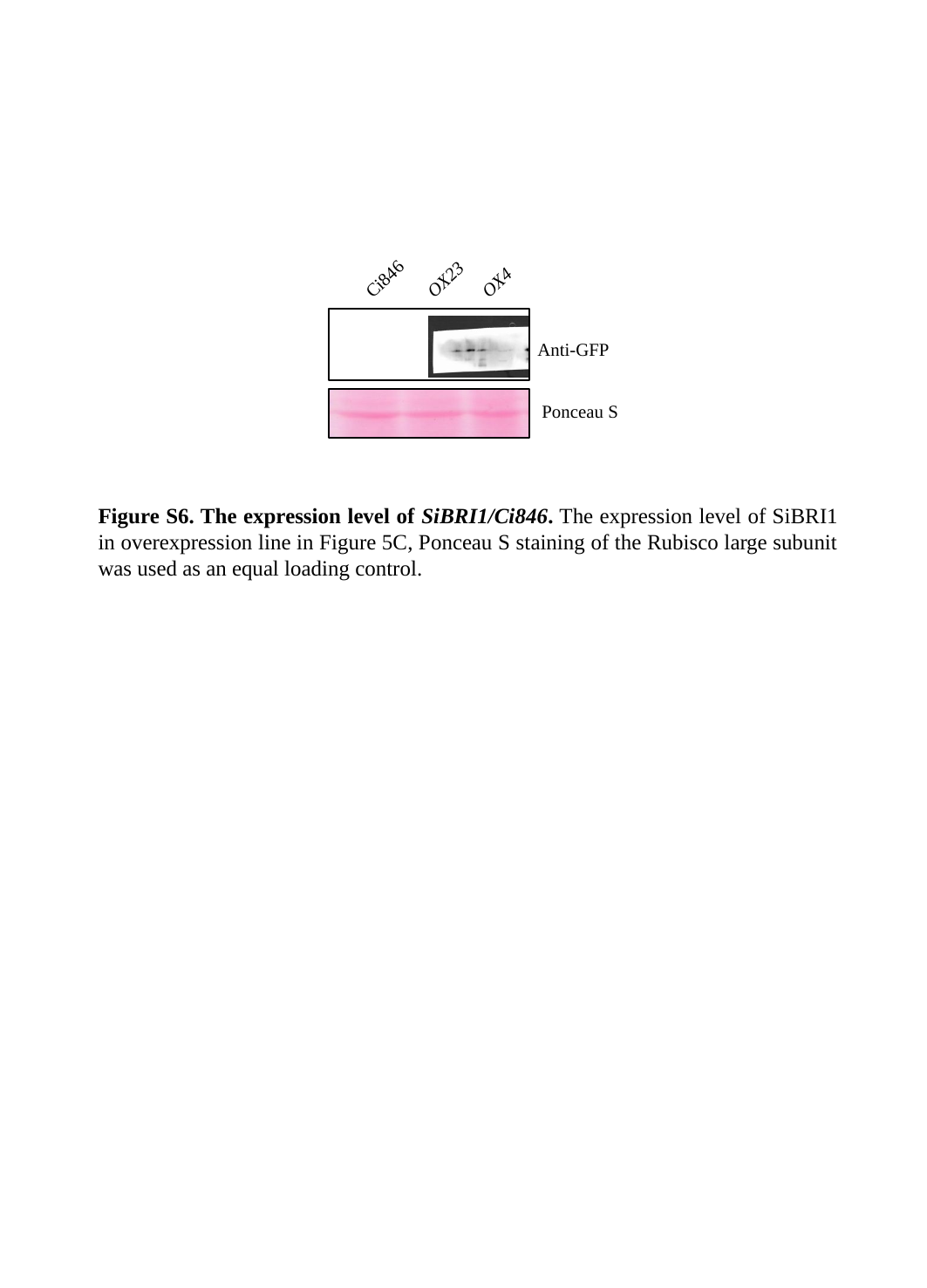

OX4
Ci846
OX23
Anti-GFP
Ponceau S
Figure S6. The expression level of SiBRI1/Ci846. The expression level of SiBRI1 in overexpression line in Figure 5C, Ponceau S staining of the Rubisco large subunit was used as an equal loading control.
