# Supplementary material for: Evolutionary analysis and functional characterization of SiBRI1 as a Brassinosteroid receptor gene in foxtail millet
Source: BMC Plant Biol. 2021 Jun 24;21:291. doi: 10.1186/s12870-021-03081-8 (PMC8223282; doi:10.1186/s12870-021-03081-8)
Supplement: Supplementary file 2 — Additional file 2. [file 12870_2021_3081_MOESM2_ESM.docx]

**Source Data Figure 2**

Merge

DIC

GFP


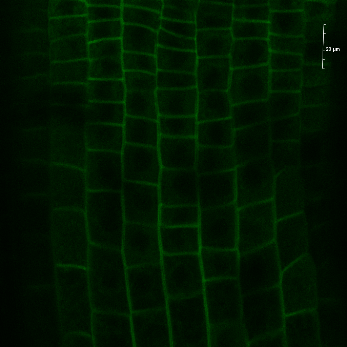

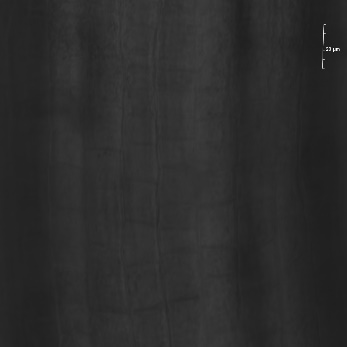

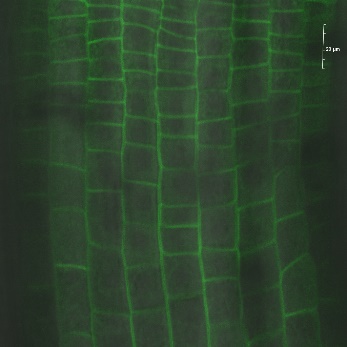


pUbi:SiBRI1-eGFP

B, Confocal images indicate the localization of SiBRI1-eGFP in the roots of 3-day-old dark-grown seedlings overexpressing SiBRI1 with a eGFP tag at the C-terminus by Olympus - Fv3000. Scale bar = 20 µm.

**Source Data Figure 3**

*35S:SiBRI1/bri1-116-7#*

*35S:SiBRI1/Col-2#*

*Col*

*bri1-116*

**B-1**

Anti-GFP





SiBRI1-YFP

130kD

170kD





HSP70

72kD

Anti-HSP70

**Short exposure**





Anti-BZR1

**Long exposure**

43kD


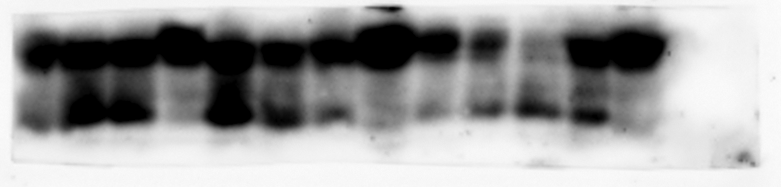


pAtBZR1

AtBZR1


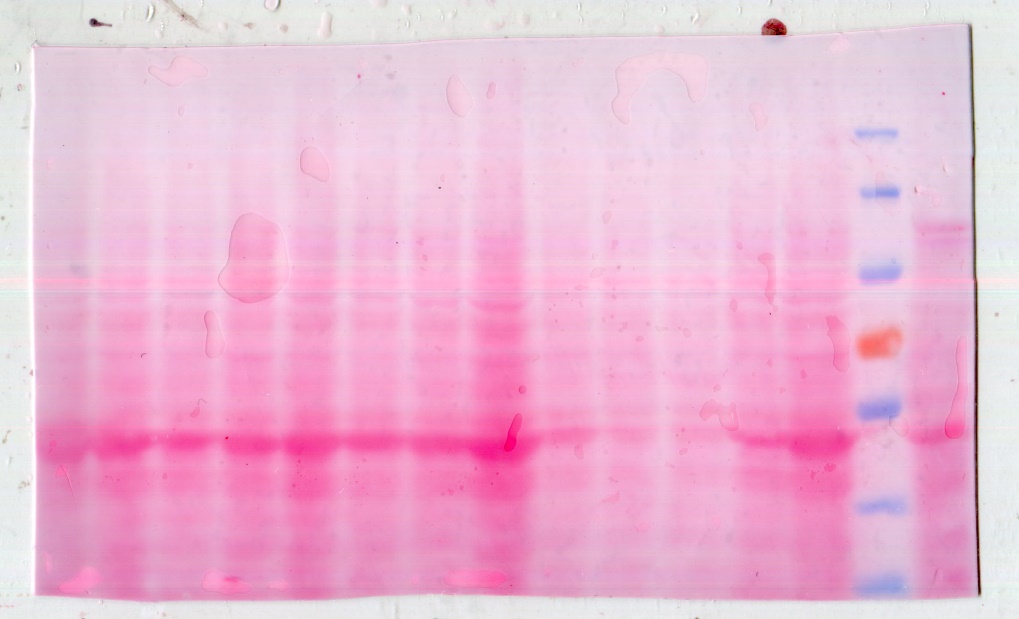


**130kD**

**95kD**

**72kD**

**55kD**

**43kD**

**170kD**

B-1, Expression levels of SiBRI1-YFP and AtBZR1 in the transgenic plants shown in Fig 3A. The differential accumulation pattern of SiBRI1-YFP was detected by anti-GFP. The differential accumulation pattern of AtBZR1 was detected by anti-BZR1; Short exposure and long exposure were shown, to clearly show the bands of pAtBZR1 and AtBZR1, we chose long exposure to Fig 3B; pAtBZR1 showed the phosphorylation form of AtBZR1, and AtBZR1 showed the unphosphorylated form of AtBZR1. Anti-HSP70 and Ponceau S staining of the Rubisco large subunit was used as an equal loading control. Ponceau S staining showed that this was one NC membrane, but in order to detect different sizes of proteins, we split it into three NC membranes and incubated them with different antibodies. The black box represents the target band. In Fig 3B, the image was flipped horizontally to match the gel image B-2.

**B-2**


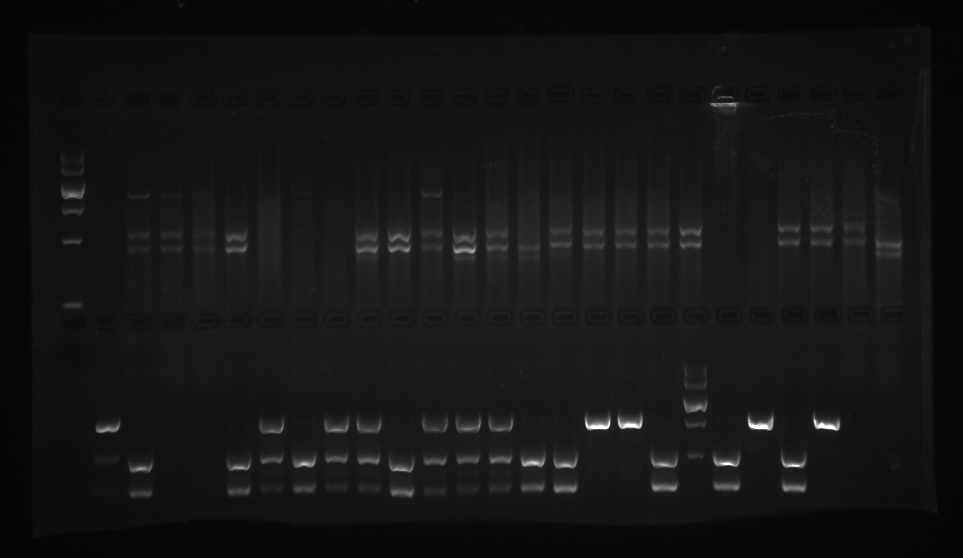


*Col*

*35S:SiBRI1/bri1-116-2#*

*35S:SiBRI1/Col-7#*

*bri1-116*

**1300bp**

**600bp**

**900bp**

B-2, The gel shows the genotyping identification of the transgenic plants in Fig 3A. The white box represents the target band.

**Source Data Figure 4**

0.25μM PCZ

*35S:SiBRI1/bri1-116-7#*

*35S:SiBRI1/Col-2#*

*35S:SiBRI1/bri1-116-7#*

*35S:SiBRI1/Col-2#*

*bri1-116*

*Col*

*Col*

*bri1-116*





HSP70

Anti-HSP70

72kD


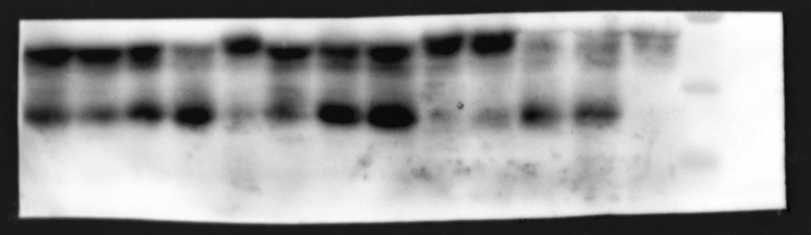


Anti-BZR1

43kD


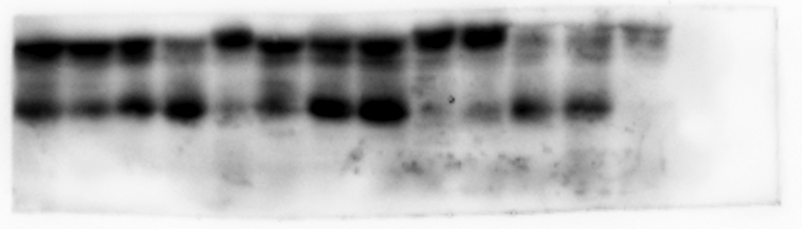


AtBZR1

pAtBZR1


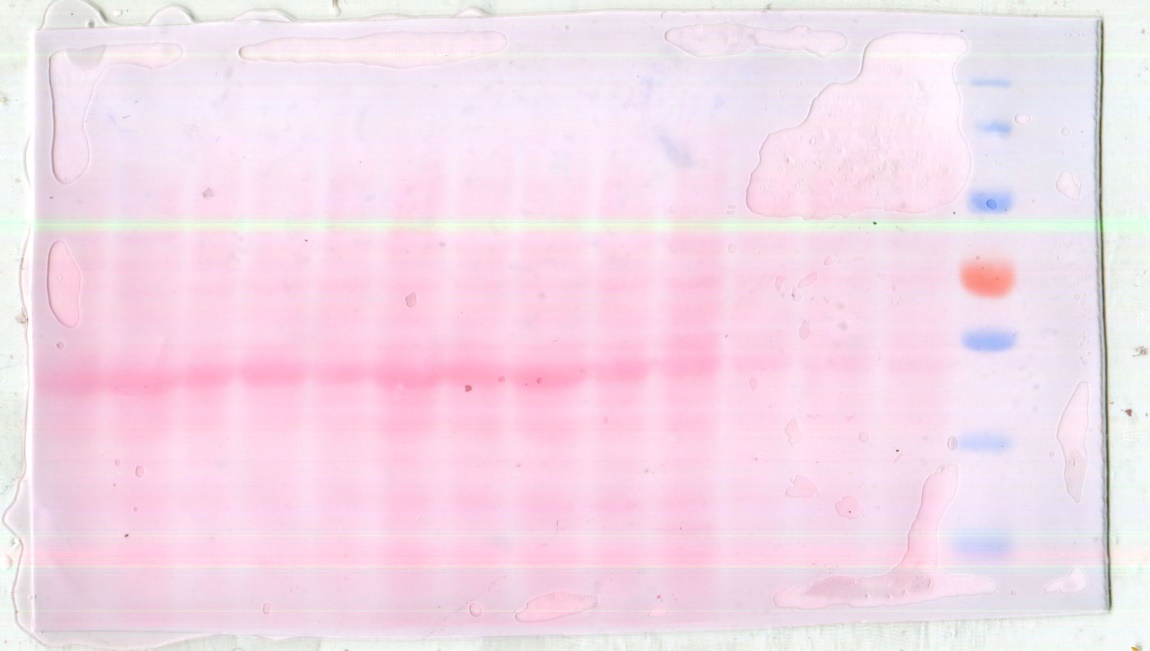


**170kD**

**130kD**

**95kD**

**72kD**

**55kD**

**43kD**

Ponceau S

E, Immunoblot analysis of AtBZR1 in transgenic plants overexpressing SiBRI1 in the *bri1-116* mutant or wild type background under PCZ. The differential accumulation pattern of AtBZR1 was detected by anti-BZR1; pAtBZR1 showed the phosphorylation form of AtBZR1, and AtBZR1 showed the unphosphorylated form of AtBZR1. Anti-HSP70 and Ponceau S staining of the Rubisco large subunit was used as an equal loading control. Ponceau S staining showed that this was one NC membrane, but in order to detect different sizes of proteins, we split it into three NC membranes and incubated them with different antibodies. The black box represents the target band.

**Source Data Figure 5**

Short exposure

*OX14*

Ci846

*OX23*





Long exposure

SiBRI1-eGFP

Anti-GFP

170kD





SiBRI1-eGFP


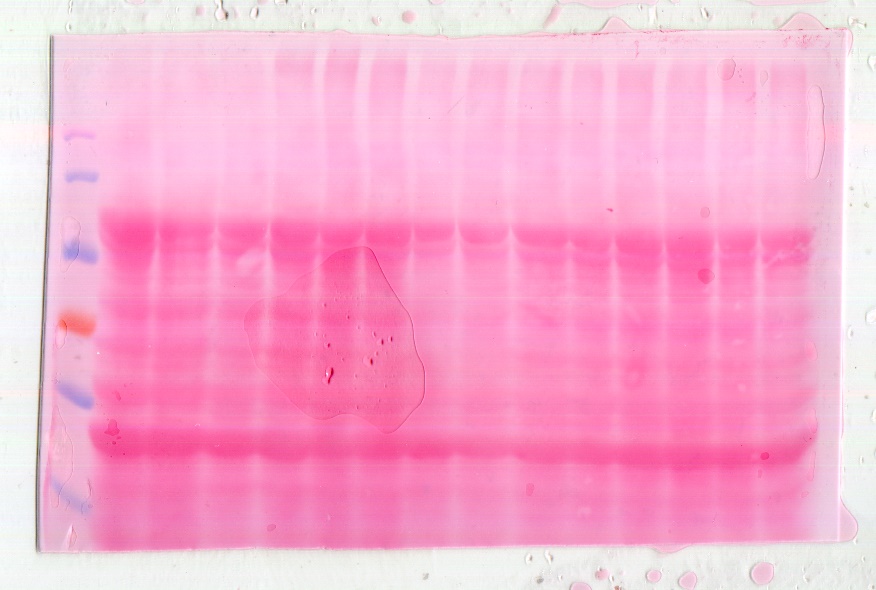


**55kD**

Ponceau S

**72kD**

**95kD**

**130kD**

**170kD**

**43kD**

B, Expression levels of SiBRI1-eGFP in the transgenic plants shown in Fig 5A. Ponceau S staining of the Rubisco large subunit was used as an equal loading control. The black box represents the target band.

**Source Data Figure S4**

*35S:SiBRI1/bri1-116-5#*

*35S:SiBRI1/bri1-116-15#*

*Col*

*bri1-116*

Anti-GFP





SiBRI1-YFP

130kD

170kD





HSP70

72kD

Anti-HSP70

Short exposure





Long exposure

43kD

Anti-BZR1


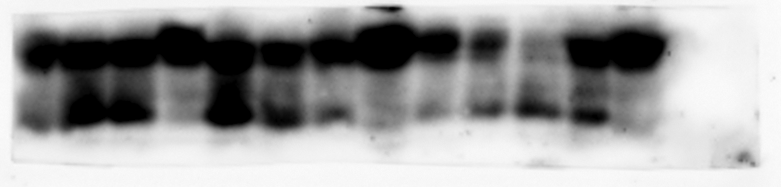


AtBZR1

pAtBZR1


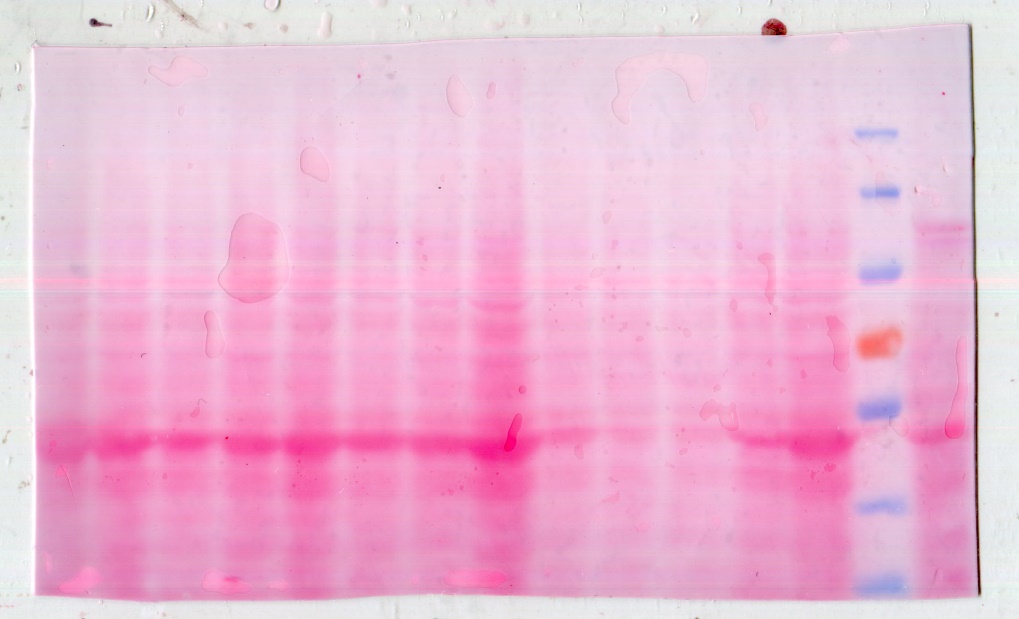


**170kD**

**130kD**

**95kD**

**72kD**

**55kD**

**43kD**

C, Expression levels of SiBRI1-YFP and AtBZR1 in the transgenic plants shown in Fig S4A. The differential accumulation pattern of SiBRI1-YFP was detected by Anti-GFP. The differential accumulation pattern of AtBZR1 was detected by anti-BZR1; Short exposure and long exposure were shown, to clearly show the bands of pAtBZR1 and AtBZR1, we chose long exposure to Fig S4C; pAtBZR1 showed the phosphorylation form of AtBZR1, and AtBZR1 showed the unphosphorylated form of AtBZR1. Anti-HSP70 and Ponceau S staining of the Rubisco large subunit was used as an equal loading control. Ponceau S staining showed that this was one NC membrane, but in order to detect different sizes of proteins, we split it into three NC membranes and incubated them with different antibodies. The black box represents the target band.

**Source Data Figure S6**

Ci846

*OX23*

*OX4*





**130kD**

SiBRI1-eGFP

Anti-GFP

**170kD**


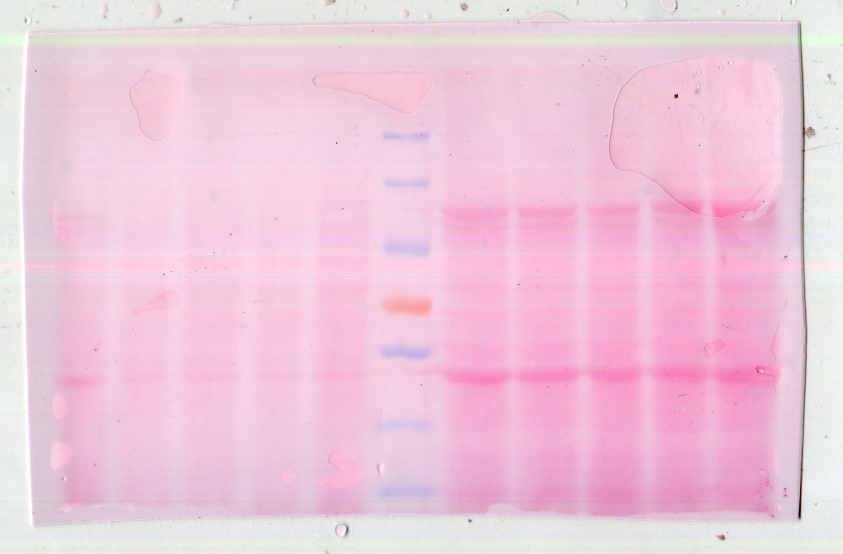


**72kD**

**43kD**

**55kD**

**170kD**

**130kD**

**95kD**

Ponceau S

The expression level of SiBRI1 in overexpression line, Ponceau S staining of the Rubisco large subunit was used as an equal loading control. The black box represents the target band.
